# Identifying and reporting modifications to surgical innovation: a systematic review of IDEAL/IDEAL-D studies

**DOI:** 10.1136/bmjopen-2024-097097

**Published:** 2025-06-30

**Authors:** James Olivier, Daisy Elliott, Kerry Avery, Natalie S Blencowe, Rhiannon Macefield

**Affiliations:** 1 NIHR Bristol Biomedical Research Centre, University Hospitals Bristol and Weston NHS Foundation Trust and University of Bristol, Bristol, Bristol, UK; 2 Leeds Institute of Emergency General Surgery, St James’s University Hospital, Leeds, UK

**Keywords:** SURGERY, Research Design, QUALITATIVE RESEARCH, Systematic Review

## Abstract

**Abstract:**

**Objectives:**

The Idea, Development, Evaluation, Assessment and Long-term follow-up (IDEAL) framework was designed to improve the quality of surgical research and evaluation of surgical innovation. It has become a widely cited tool for evaluating innovative devices and procedures, yet challenges remain concerning the definition and reporting of incremental innovative modifications, hindering evolution and evaluation of innovations and potentially risking patient safety. This systematic review examined IDEAL studies to identify such modifications and establish recent practices around modification reporting to inform the development of future guidance to facilitate safe, transparent and efficient surgical innovation.

**Design:**

Systematic review and thematic synthesis of studies reporting surgical innovation.

**Data sources:**

Web of Science and Scopus were searched in July 2023 using citation tools to identify studies following the IDEAL framework (citing any of 13 key IDEAL/IDEAL framework publications and guideline papers).

**Eligibility criteria:**

Primary research studies of any design that involved invasive innovative devices or procedures.

**Data extraction and synthesis:**

Study characteristics and verbatim text for all reported modifications, including contextual information, were extracted. Data were analysed and synthesised using thematic synthesis.

**Results:**

Of 1071 records screened, 104 studies published between 2011–2023 were included (n=87 (83.6%) study reports; n=17 (16.3%) protocols). 425 modifications were reported in 76 (73.1%) studies, including modifications to procedures (n=283, 66.6%), devices (n=94, 22.1%) and patient selection (n=48, 11.3%). Procedure/device modifications included technical, non-technical and cessation (conversion to other procedures or abandonment). Modifications were most often reported within IDEAL stage 2a (n=30/44, 68.2%), whereas there was considerable variation across other stages, such as stage 0 (n=2/3, 66%) and stage 2b (n=4/12, 33.3%).

**Conclusion:**

Reporting modifications is imperative for evaluating surgical innovation. However, this review found inconsistent approaches to reporting and describing modifications. Findings will inform the development of a checklist for reporting modifications that aims to complement the IDEAL framework and further promote shared learning, avoiding the repetition of harmful/ineffective modifications and enhancing patient safety.

**PROSPERO registration number:**

CRD42023427704.

**STRENGTHS AND LIMITATIONS OF THIS STUDY**

- A wide-ranging scope for eligible studies enabled the inclusion of a broad range of innovative devices and procedures across multiple stages of innovation and specialties.
- A comprehensive spectrum of modification types was extracted to ensure that all information potentially relevant to the evolution of surgical innovations was captured.
- Two source databases with the functionality to perform and export citation searches were used, meaning potentially relevant studies not indexed in these databases may have been missed.
- Searches for relevant studies were limited to English-language publications citing the Idea, Development, Evaluation, Assessment and Long-term follow-up framework.

## Introduction

 Over 10 million surgical procedures are undertaken every year in the UK, with forecasted costs estimated to be close to £9.5 billion.^1 2^ Surgical innovation is critical to advance treatments and improve patient outcomes, yet it often lacks rigorous evaluation. This can mean potentially harmful procedures, devices and techniques become widespread, before their risks are recognised. High-profile examples with disastrous consequences for patients include silicone breast implants,^3^ vaginal mesh^4^ and metal-on-metal hip replacements.^5^ A 2020 independent safety review led by Baroness Cumberlege identified fundamental failings in existing mechanisms for evaluating surgical innovation and recommended robust evaluation in line with the scrutiny applied to pharmacological interventions.^6^ This, however, is challenging because surgical innovation occurs very differently from the development and evaluation of new drugs. Surgical innovations, for example, may often undergo incremental and iterative modification (for example, refinements or changes in technique) by surgeons as they perform more cases. Many of these modifications are not documented, reported or shared, as current evaluation systems are not designed to capture such changes.

The Idea, Development, Exploration, Assessment, Long-term follow-up (IDEAL) recommendations, introduced in 2009 to improve the introduction and evaluation of innovative surgical procedures,^7^ recognise the above challenges. Further updates have since been published to provide practical guidance on applying IDEAL in practice,^8 9^ yet while use of the framework has increased, there have been challenges in widespread adoption.^9^,^15^ The IDEAL framework suggests that modifications tend to occur in the development stage before an innovative procedure reaches stability, yet it also states that modifications can occur in the exploration stage as the procedure is approaching readiness for definitive evaluation.^16^ In comparison, the IDEAL guidelines for device evaluation (IDEAL-D) suggest that most modifications occur in a preclinical stage.^17 18^ The IDEAL framework, therefore, highlights the importance and benefit of reporting modifications, but practical guidance and the conceptualisation of both modifications and stability are lacking.^19^ If modifications cannot be identified and reported, the point at which an innovation stops evolving and becomes sufficiently stabilised for definitive evaluation may be difficult to recognise.

There is currently no widely accepted definition for modifications in surgical innovation,^20^ and different terms have been used interchangeably in the literature, such as “variations”, “refinements”, “tinkering” or “alterations”.^21^,^23^ This contributes to a heterogeneous and inconsistent approach to reporting surgical innovation research and evaluating new surgical procedures and devices, both being recognised problems in the existing literature.^24 25^ Relevant literature suggests that modifications likely cover a broad spectrum of changes, such as changes to the technical components of a procedure or device, to co-interventions (ie, interventions that occur before, during or after the primary intervention under evaluation)^26^ and to the characteristics of patients who are offered the intervention.^20 27^ Decisions to make modifications are often based on individual surgeons’ reflections and experiences of using an innovation in clinical practice. These decisions and practices, however, can be siloed and modifications may not be well-documented or shared with other surgeons.^28^ This creates a lack of transparency surrounding ‘successful’ and ‘unsuccessful’ modifications, hampers efficient evaluation and creates variation between surgeons and centres. Importantly, this can potentially perpetuate patient harm by inadvertently repeating modifications that can negatively impact patient outcomes. Work to understand modifications in more detail and how best to report them is needed. A review of surgical innovation studies focusing on modification reporting has not yet been undertaken. This systematic review examined the recent practices for reporting modifications in studies following the IDEAL/IDEAL-D framework to inform the development of guidance for identifying and reporting modifications in surgical innovation.

## Methods

A study protocol was drafted a priori and prospectively registered on the PROSPERO database (CRD42023427704).^29^ This study is reported according to Preferred Reporting Items for Systematic reviews and Meta-Analyses (PRISMA) recommendations.^30^

### Search strategy

Our strategy to identify studies that followed the IDEAL/IDEAL-D framework involved two steps. First, electronic searches were undertaken to identify studies that cited any of the 13 key IDEAL/IDEAL-D framework publications and guideline papers.^79 16^,^18 23 31^ Searches were performed in Scopus and Web of Science databases because they have superior functionality for thorough and accurate citation searches (eg, identifying records that have cited any of the 13 key IDEAL/IDEAL-D studies) than other databases.^38 39^ Date limitations to search for publications from 2019 onwards were applied, as studies published before this date were sourced from an existing review of IDEAL/IDEAL-D studies.^40^ Searches were performed in July 2023, and results were migrated into V.20 of EndNote.^41^ Second, records were reduced to only select those that had included the words ‘IDEAL’ or ‘IDEAL-D’ in the title or abstract. This approach for identifying and focusing on IDEAL/IDEAL-D studies as the foundation for this review was based on the rationale that authors of studies aligned to the IDEAL framework are more likely to have considered the importance of reporting modifications, providing a suitable data source for examining recent practices. This strategy to identify IDEAL/IDEAL-D studies was based on an existing review of outcome measurement and reporting in IDEAL-cited studies published in 2020.^40^ Further details related to the search strategy can be found in online supplemental file 1.

### Study selection

Eligible publications for inclusion were primary research studies involving innovative, invasive procedures and devices.^40^ A pre-existing definition of invasive procedures was used: ‘purposeful or deliberate access to the body gained via an incision, percutaneous puncture, where instrumentation is used in addition to the puncture needle or instrumentation via a natural orifice’.^42^ Studies in which innovative invasive procedures/devices were co-interventions (eg, radiological imaging for radiologically guided invasive biopsy) were also included due to their potential value in identifying modifications relevant to evaluating surgical innovation. Systematic reviews, book chapters, letters, commentaries, conference proceedings, technical notes, abstracts, editorials and non-English-language publications were excluded.

Records were exported into electronic software (Rayyan)^43^ to facilitate the screening process. Screening of titles and abstracts for potential eligibility was undertaken by one reviewer (JO), with 10% of records independently screened by a second reviewer (RM) for validation.^44^ This sample was selected randomly by alphabetising the records by title and selecting every 10th study. Next, full texts of potentially relevant records were obtained via online sources or by contacting authors. Two independent reviewers (JO and RM) screened all full texts to confirm inclusion. Any discrepancies were discussed and resolved with the wider study group. All full-text papers identified from the screening process were supplemented with studies identified in an existing review of IDEAL/IDEAL-D studies that focused on outcome reporting.^40^ This provided a comprehensive data source of IDEAL/IDEAL-D studies published since the establishment of the IDEAL framework in 2009.

### Data extraction and analysis

A standardised data extraction form was developed and piloted by the study team and used for secure electronic data capture and storage in REDCap software.^45^ One reviewer (JO) extracted data chronologically for each publication, from the newest study to the oldest. The review team held regular meetings to ensure agreement on the approach to data extraction and the data types extracted.

#### Study characteristics

Details of the innovation, year of publication, number of participating centres and surgeons, number of patients and geographical origins of the study were extracted.

#### IDEAL stages

The IDEAL stage or stages allocated to studies by authors were extracted. If the authors did not state a specific IDEAL stage, reviewers collectively allocated an IDEAL stage or stages to the study based on a published decision aid to help identify the IDEAL stage of innovation from literature reports.^9^

#### Modifications

Any details on modifications, including those relevant to the technical components of the procedure or device, to co-interventions or to patients selected for the procedure, were extracted verbatim. Any contextual information or stated rationale relevant to these modifications was also extracted.^46^ All verbatim data relating to modifications were then exported from REDCap into NVivo V,12 Pro.^47^ The Review question, Epistemology, Time/Timescale, Resources, Expertise, Audience and purpose, Type of data criteria^48^ identified that thematic synthesis would enable the categorisation of findings and subsequent development of themes (patterns in the data).^49^ This involved line-by-line coding, where text segments were systematically assigned codes (eg, ‘labels’). These were iteratively developed as the analysis progressed until no further themes were identified (eg, saturation of thematic synthesis was achieved). Throughout this process, the primary reviewer (JO) met regularly with the review team to reflect on analysis findings and ensure consistency in approach.

### Patient and public involvement

The concept of this research was informed by a public engagement event (FUTURES Festival of Discovery, UKRI funded) held on the SS Great Britain, Bristol. Here, members of the public participated in conversations around innovation in surgery and the role of modification identification and reporting in this process. They also interacted with a life-size ‘Operation Game’ (Altitude Events, Felbridge, UK) and written materials around the work undertaken in the Bristol Biomedical Research Centre. Members of the public highlighted the importance of transparency around modifications to innovative devices and procedures with foreseeable benefits in safety and efficiency of innovation. The review is also part of broader work within the National Institute for Health and Care Research Bristol Biomedical Research Centre Surgical Innovation theme, which regularly undertakes patient and public involvement and engagement activities around surgical innovation to develop ideas, consult on study design and seek advice. Due to its methodological focus, patients or members of the public were not directly involved in this systematic review’s data collection or analysis.

## Results

**Figure 1:**
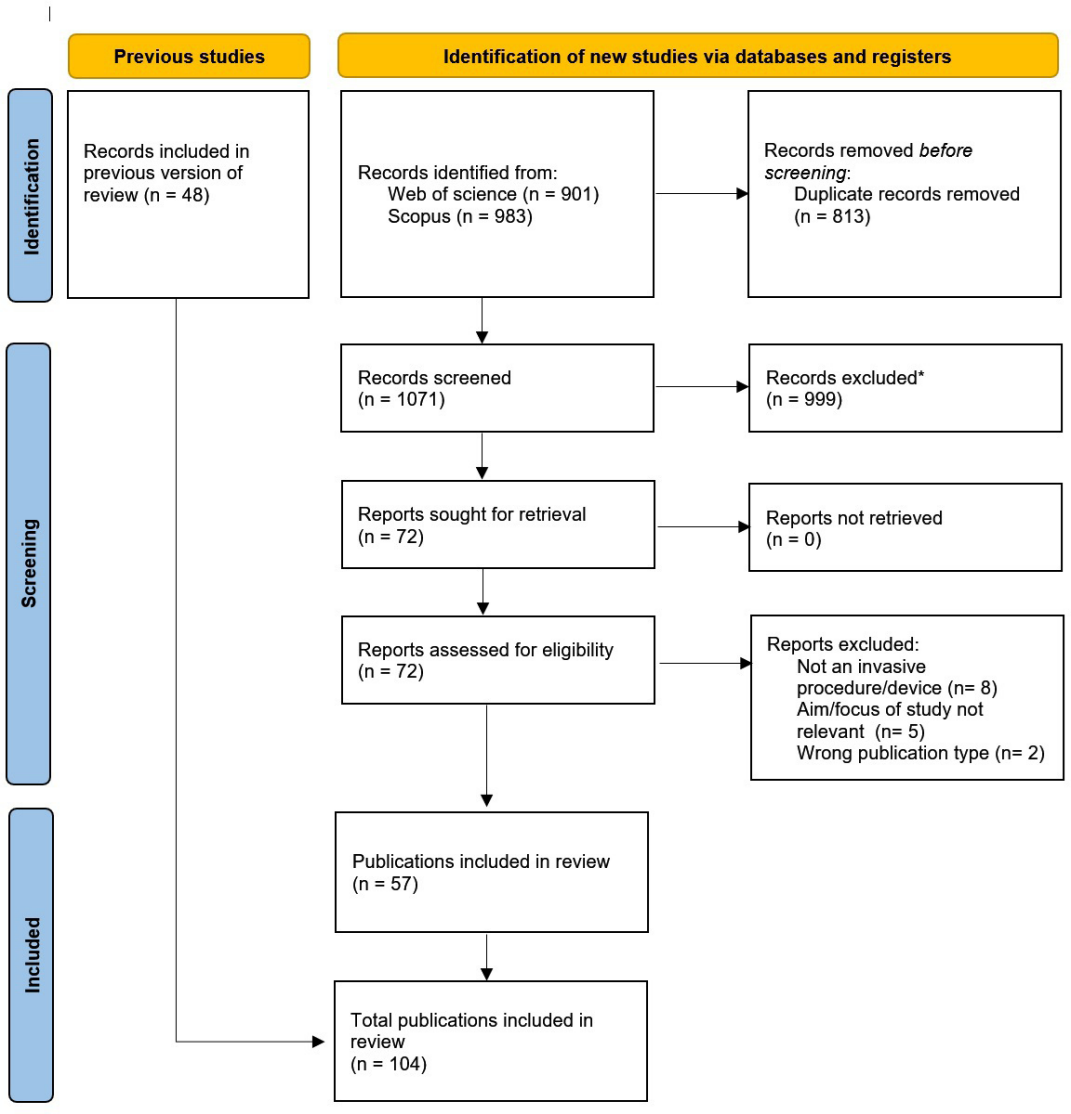
PRISMA diagram showing identification, screening and inclusion of studies for review. *Reasons for exclusion (999 studies): not including IDEAL/IDEAL-D, wrong publication type or not an invasive procedure/device. 1 record was duplicated between the previous review and this current review therefore resulting in 104 total included publications (not 105). IDEAL, Idea, Development, Exploration, Assessment, Long-term follow-up; IDEAL-D, IDEAL guidelines for device evaluation; PRISMA, Preferred Reporting Items for Systematic reviews and Meta-Analyses.

Database searches identified 1071 unique records, with 157 (14.6%) containing the words ‘IDEAL’ and/or ‘IDEAL-D’ in the title and/or abstract. Following abstract screening, 72 records (45.9%) were identified as potentially eligible and proceeded to full-text review. About 57/72 studies (79.2%) met the eligibility criteria for inclusion after full-text review. Reasons for exclusion are listed in the PRISMA diagram^30^ (figure 1). Combined with 48 studies identified in the existing review of IDEAL/IDEAL-D studies,^40^ 104 publications were included for data extraction and analysis (see online supplemental file 2 for the complete list of citations). Given the exploratory nature of this review, a risk of bias or quality assessment for the included studies was not undertaken, similar to that of other exploratory reviews of surgical innovation studies.^40 50^

### Study characteristics

Table 1 summarises the key characteristics of all 104 included studies, published between 2011–2023. A detailed description of study characteristics is provided in online supplemental file 2. Study reports made up 87 (83.7%) of the publications and 17 (16.3%) were protocols. Case series (n=62, 59.6%) and cohort studies (n=22, 21.2%) were the most frequent study design types, whereas only six (5.8%) were randomised controlled trials. The most common innovation types reported were procedures (n=71, 68.3%) and devices (n=24, 23.1%). These innovations arose from 11 surgical specialities, with urology being the most highly represented (n=38, 36.5%). The number of centres included per study varied from 1 to 40, with a wide range of study sizes (1–2322 participants). In 45 (43.3%) studies, the number of surgeons/operators was not reported, but the range was 1–28 in studies providing this information.

**Table 1:** Characteristics of included publications

		Total number of included studies (%)	Number of studies categorised by IDEAL stage						
			0 (n=3)	1(n=18)	2a (n=41)	2b (n=12)	3(n=5)	4 (n=1)	Multiple or unable to stage (n=24)
Study design	Case series	62 (60)	–	12	35	2	2	1	10
	Case study	5 (5)	–	5	–	–	–	–	–
	Cohort study	22 (21)	–	1	5	9	–	–	7
	Multiple study designs	6 (6)	–	–	–	–	–	–	6
	Preclinical study	3 (3)	3	–	–	–	–	–	–
	Randomised trial	6 (6)	–	–	1	1	3	–	1
Surgical specialty	Breast surgery	5 (5)	–	–	2	–	–	–	3
	Cardiothoracic Surgery	1 (1)	–	–	1	–	–	–	–
	Colorectal surgery	12 (12)	–	1	6	–	–	–	5
	Head and Neck	5 (5)	–	2	3	–	–	–	–
	Hepatobiliary	8 (8)	1	2	4	–	1	–	–
	Multiple	2 (2)	–	–	2	–	–	–	–
	Neurosurgery	2 (2)	–	1	–	–	–	–	1
	Obstetrics and gynaecology	11 (11)	–	4	1	4	1	–	–
	Oesophagogastric	7 (7)	–	–	3	1	–	–	3
	Orthopaedics	5 (5)	1	–	2	–	–	–	2
	Paediatric Surgery	1 (1)	–	1	–	–	–	–	–
	Transplant	8 (8)	–	2	1	1	–	–	4
	Urology	38 (36)	1	5	17	6	3	1	5
Country	European Economic Area (Non-UK)	52 (50)	1	10	22	6	3	1	8
	Multiple	11 (11)	–	1	3	1	1	–	5
	North America	7 (7)	–	3	2	–	–	–	2
	Other	15 (14)	–	1	6	5	–	–	3
	UK	19 (18)	2	3	8	0	1	–	5
Publication year	2011–2014	10 (10)	–	2	3	–	8	–	5
	2015–2018	36 (35)	–	7	14	8	1	–	5
	2019–2023	58 (56)	3	9	24	4	4	1	13
Single or multicentre study	Single centre	76 (73)	3	17	32	7	2	1	13
	Multicentre	20 (19)	–	–	4	5	2	–	7
	Not reported	8 (8)	–	1	4	–	–	–	3
Number of surgeons performing the intervention	1–2	39 (38)	2	8	14	2	2	–	10
	3–7	16 (15)	0	1	8	3	–	–	4
	>8	2 (2)	1	–	–	–	–	1	–
	Not reported/unclear	47 (45)	–	9	18	7	3	–	9
Number of participants	1–10	21 (20)	1	12	5	–	–	–	3
	11–50	51 (49)	2	6	31	3	–	–	8
	51–100	13 (13)	–	–	4	2	3	–	4
	101–400	14 (13)	–	–	1	5	1	–	7
	>400	5 (5)	–	–	–	2	1	1	1

IDEAL, Idea, Development, Exploration, Assessment, Long-term follow-up.

### IDEAL stages, use and interpretation

The number of studies with a single allocated IDEAL stage was 73 (70.2%). This was most commonly IDEAL stage 2a (development), in 41 (39.4%) studies (table 1). 22 (21.2%) studies reported innovations that spanned multiple IDEAL stages. The authors of nine studies (8.7%) did not specify an IDEAL stage. For these studies, the authors of this review collectively determined the IDEAL stage, except for one study that contained insufficient information and was labelled ‘unable to stage’.

**Figure 2:**
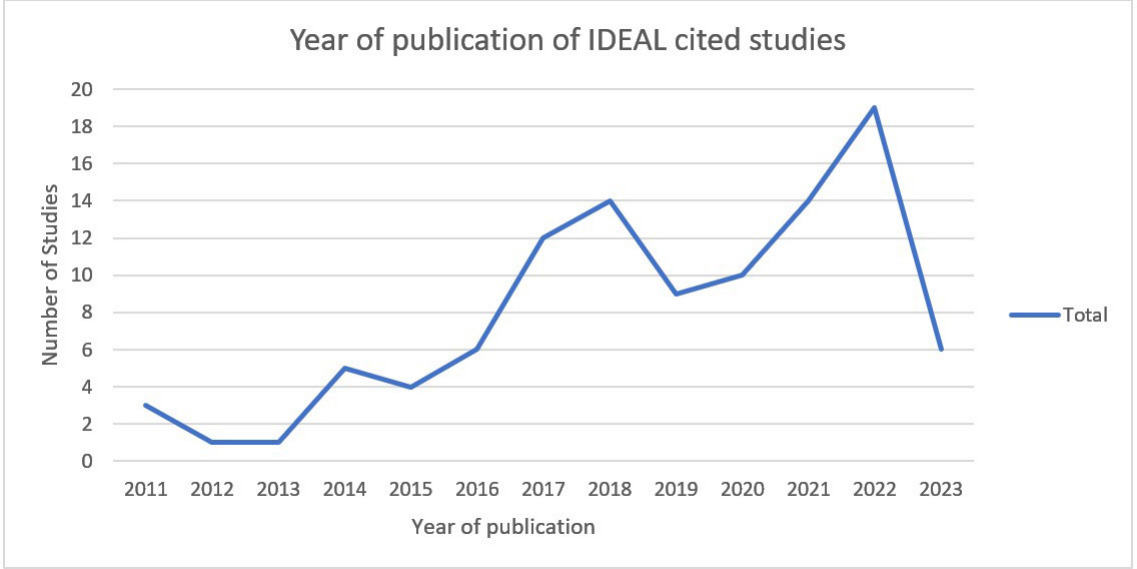
Number of studies citing IDEAL over time (up to July 2023). IDEAL, Idea, Development, Exploration, Assessment, Long-term follow-up.

The number of included studies citing IDEAL increased over time (figure 2). Most studies aligned closely with IDEAL recommendations and expectations for the various stages of innovation regarding the number of patients, centres and surgeons involved in delivering an innovation. However, there were exceptions, with some studies involving more patients, centres or surgeons than usual for the IDEAL stage of innovation, such as one author-reported stage 1 (idea) study involving 37 patients. Another study reported an initial ex-vivo simulation as stage 1, the first three patients as stage 2a and the subsequent 15 patients as stage 2b. The IDEAL recommendations would typically define these instead as stage 0, stage 1 and stage 2a, respectively. Of the 24 studies reporting on innovative devices, only seven (29.1%) cited the device-specific IDEAL-D framework, whereas 17 (70.9%) solely cited the procedure-specific IDEAL framework, although two (8.3%) of these were published before the issuance of IDEAL-D guidance.

### Modification reporting

#### Incidence/frequency of reporting

**Figure 3:**
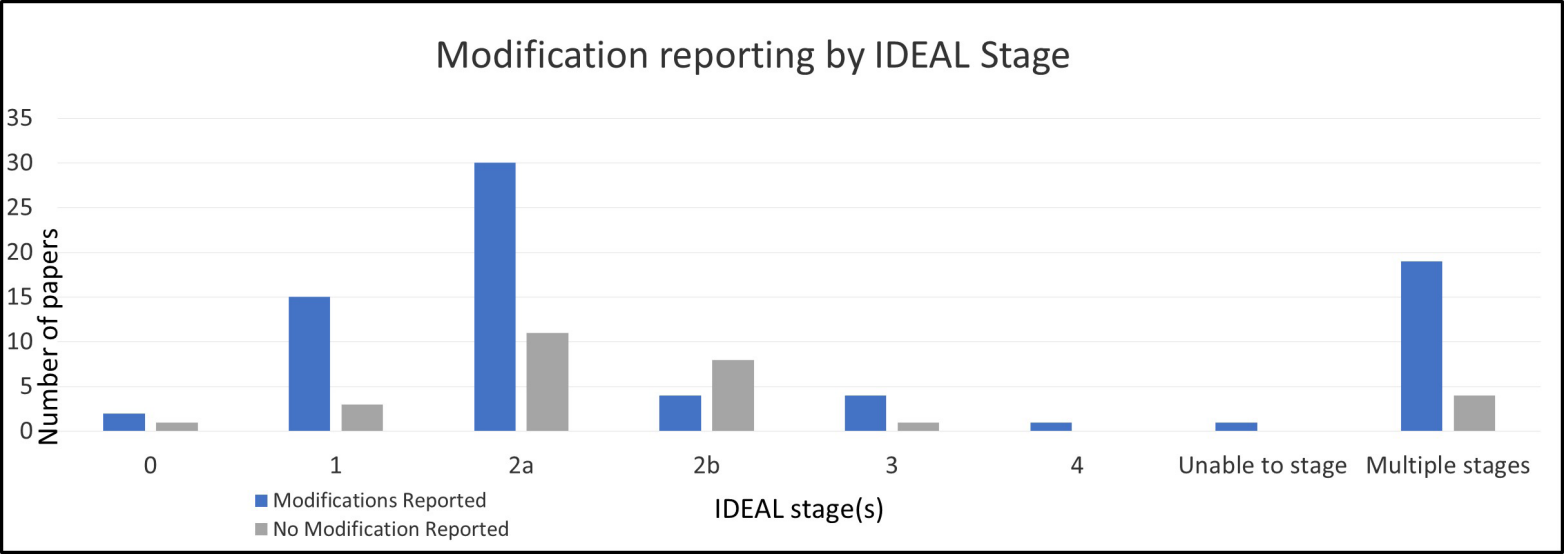
Number of studies reporting any modifications by IDEAL stage. IDEAL, Idea, Development, Exploration, Assessment, Long-term follow-up.

Of the 104 studies included, 76 (73.1%) reported modifications. Full details of studies that did and did not report modifications (with citations) are provided in online supplemental file 2. Although modifications were reported in studies across all IDEAL stages (figure 3), they were most commonly reported in stage 2a studies (n=30/104, 28.8%). In all stages, proportionally more studies reported modifications than those that did not, except for stage 2b (figure 3). Modifications were presented graphically (such as in a table, graph or other visual representation) in 24 (23.1%) studies. Some studies reported modifications solely or predominantly in the online supplemental files instead of within the main paper.

#### Types of modifications reported

Across the 76 studies reporting modifications, the total number of individual modifications identified and extracted from the studies was 425. The number of modifications reported in a single study ranged from 1 to 19. Most modifications related to surgical procedures (n=283, 66.6%), compared with 94 (22.1%) modifications to devices and 48 (11.3%) modifications to patient selection criteria (table 2). Detailed qualitative analysis (ie, coding) of the extracted modification data identified further subcategories of types of modifications. Modifications were categorised within the procedure and device categories as technical, non-technical and cessation (ie, conversions or abandonments). Technical modifications were the most common type of modifications in each category (n=225 for procedures, n=92 for devices). Technical procedural modifications included changes, additions, repetitions or removals of specific procedure steps, changes in a procedural co-intervention or co-treatment (such as anaesthetic agents), changes in the materials or tools used for the procedure and changes in patient position or approach. Technical device modifications included changes to the components of a device, the number of devices used, the size of a device or how a device is used/applied/inserted. Non-technical modifications included alterations in expertise, personnel or non-technical skills. Modifications to patient selection criteria included changes to inclusion and exclusion criteria across both devices and procedures.

**Table 2:** Number and type of modifications across all studies reporting any modifications

Modification category/themes derived from the data		Number of reported modifications (n=425)
Procedural		Total=283 (66.6%)
	Technical	225 (79.5%)
	Non-technical	4 (1.4%)
	Conversions or abandonments	54 (19.1%)
Device		Total=94 (22.1%)
	Technical	92 (97.9%)
	Non-technical	0 (0%)
	Conversions or abandonments	2 (2.1%)
Patient selection criteria		Total=48 (11.3%)

#### Contextual information

Modifications were often described in the context of the timeframe of the innovation, with some occurring before the current study (ie, in a previous study/use of the procedure or device), some during the study and some proposed as future modifications. The intentionality of modifications was described in six (5.8%) studies, either as preplanned or spontaneous/unplanned (eg, in response to an unexpected event during surgery). Modifications were described in terms of magnitude in only two (1.9%) studies, in which authors used the terms *major* and/or *minor*. 44 (42.3%) studies included implications of modifications (ie, any benefits and/or drawbacks).

#### Language around modification reporting

Examples of non-specific or obfuscating language used around modification reporting were identified across the included papers. For example, it was sometimes unclear if a modification had occurred or was theorised as a hypothetical solution to a potential problem. In some studies, the presence of modifications was alluded to but without sufficient detail to allow the reader to learn from it, such as, *‘ The intervention needed to be refined’* or *‘With the refinement of the technique and the rising learning curve, these side events did not occur again’*. A wide array of alternative terms for modifications or the process of modifying an innovation were identified across the study reports, although none of the studies included definitions for any of these terms. Examples included *adaptations, additions, adjustments, alterations, changes, choices, combinations, developed, expanded, evolve, fine-tune, optimise, improvements, increments, introduced, optimised, refinements, replacing, reverted, solutions* and *substituted*.

## Discussion

This review examined primary literature around surgical innovation, focusing on understanding recent reporting practices of modifications to innovative procedures and devices. Within the 104 included studies, wide heterogeneity in the study methods, patient numbers and level of modification reporting was observed. Over a quarter of the included studies did not report or contain any mention of modifications. Where modifications were reported, this occurred across all IDEAL stages but were most frequently in IDEAL stage 2a studies (n=30, 28.8%). There was a preponderance of technical (as opposed to non-technical) modifications; however, the depth and breadth of modification reporting varied noticeably. Some authors reported multiple modifications in detail and included text about the benefits of doing so (such as transparency and shared learning), yet modification reporting itself was inconsistent.

The IDEAL framework recognises the importance of reporting modifications, particularly in stage 2a, but lacks clarity over how this should be done in practice.^7 17^ This emphasis on modification reporting in early innovation may be reflected in the frequency of modification reporting in stage 2a studies within this review. The observed variation in modification reporting in other IDEAL stages may be partly due to a lack of guidance on identifying and reporting modifications in surgical innovation, particularly in non-stage 2a studies.^19^ The studies included in this review demonstrated inconsistent approaches to implementing and using IDEAL guidance across all stages, which aligns with findings from previous reviews on the uptake of the IDEAL framework.^12 51^ This review and qualitative analysis of primary surgical innovation research complements the findings of a scoping review of modification discussions in secondary research (opinion pieces and reviews) across 49 studies up to October 2020.^20^ Overlap in some conceptual themes was seen, particularly around the contextual details of modifications (including what, when, why and so what).^20^

Detailed qualitative analysis of the extracted data around modifications and their contextual details identified some studies with particularly rich reporting of modifications.^52^,^55^ These studies often contained strategies for identifying modifications, such as prospective data capture on a case-by-case basis and using embedded qualitative methods, such as interviewing the users of an innovation after each case or examining their written journals/materials (eg, operation notes). This allows the recording of detailed contextual details around modifications, including what was changed, when modifications occurred (ie, which patients), why they were implemented (ie, the rationale) and their impact (ie, the benefits and drawbacks). Reporting these details may help to facilitate shared learning between innovators and the surgical/research community by providing a more detailed understanding of the modifications and their implications for the evolution and evaluation of the innovation. At times, the presentation of these contextual details was optimised with graphical representations of modifications and the use of the prospective development study format advocated for stage 2a studies by the IDEAL framework.^9 35^ When reporting multiple IDEAL stages, some authors specified which modifications occurred within each specific stage, either described throughout the study or as a summary with key learning points in the discussion section. Some authors reflected on the iterative nature of innovation and the role of modifications concerning stability and the learning curve. In commenting on the process of reaching stability (ie, when the frequency of modifications is reduced), the context of the modifications and the innovation is set for the reader. Using these strategies when identifying, evaluating and reporting modifications to surgical innovations could significantly contribute to addressing the failures identified in the Cumberlege safety report.^6^

To our knowledge, this is the first systematic review of primary literature around surgical innovation that has examined the reporting of modifications. The 104 studies covered all stages of innovation, including multiple types of innovations published across 12 years, providing sufficient information to establish reporting practices across the literature via a representative sample. Additionally, this study adopted a broad inclusion strategy for identifying any modifications to devices, procedures or patient selection criteria, which was undertaken based on a lack of an accepted definition for modifications.^20^ Despite these strengths, potential limitations must be recognised. Only two databases have the functionality required for reliable citation searching^38 39^; therefore, studies not indexed in these databases could have been missed. Non-English language publications and grey literature were not included for pragmatic reasons due to resource limitations. Therefore, some potentially relevant studies could have been missed. Only studies that cited IDEAL/IDEAL-D and included these words within the title and/or abstract were included. This strategy was employed as it was felt that those authors actively engaging with and using current best practices were most likely to include thorough modification reporting. It is acknowledged that studies following the IDEAL framework but not specifying this in the abstract/title or those not citing IDEAL at all would not have been identified by the current search strategy, which might introduce an element of bias. The original IDEAL framework was published in 2009^7^; therefore, studies published pre-2009 were not included. However, the authors believe that reporting standards are likely to have improved since the publication of the IDEAL framework compared with more historical publications. The final limitation is that this review does not contain any studies after July 2023 (when data searches were conducted).

This review has found that modification reporting is inconsistent and unreliable in surgical innovation research, particularly in non-stage 2a studies. Developing a tool for identifying and reporting modifications has the potential to improve shared learning, enhance the evaluation and implementation of surgical innovation and reduce patient risks. The findings from this review and analysis are currently being used to develop a checklist that will allow surgeons to identify and report modifications across all stages of surgical innovation in a systematic, pragmatic and standardised fashion. This checklist will be able to be used alongside the IDEAL framework and complement conceptual work around modifications.^20^ The detailed qualitative findings from this review will be presented in future publications when synthesised and combined with findings from subsequent indepth stakeholder interviews. It has previously been shown that surgeons often struggle to distinguish between innovation and variation, which has facilitated work on defining surgical innovation.^25^ A tool has been developed to define innovation and enable surgeons to identify innovations and distinguish them from variations (or modifications).^24 56^ The checklist that is being developed from this review will complement this tool by clarifying how to identify and report modifications. It will also enable the operationalisation of the 2023 COHESIVE core outcome set (a consensus agreed set of outcomes to measure and report, as a minimum, in all early-phase studies evaluating novel surgical procedures/devices) in which modifications were one of the eight core domains in the agreed core set.^27^

## Supplementary material

10.1136/bmjopen-2024-097097 online supplemental file 1

10.1136/bmjopen-2024-097097 online supplemental file 2

## Data Availability

Data are available upon reasonable request.

## References

[1] Abbott TEF, Fowler AJ, Dobbs TD (2017). Frequency of surgical treatment and related hospital procedures in the UK: a national ecological study using hospital episode statistics. Br J Anaesth.

[2] FTN (2014). Briefing: operating theatres - maximising a valuable resource 2014. https://nhsproviders.org/media/1128/operating-theatres-final.pdf.

[3] Greco C (2015). The Poly Implant Prothèse breast prostheses scandal: Embodied risk and social suffering. Soc Sci Med.

[4] Heneghan C, Aronson JK, Goldacre B (2017). Transvaginal mesh failure: lessons for regulation of implantable devices. BMJ.

[5] Cohen D (2012). How safe are metal-on-metal hip implants?. BMJ.

[6] Medicines I (2020). Medical devices safety review: first do no harm—the report of the immdsreview.

[7] McCulloch P, Altman DG, Campbell WB (2009). No surgical innovation without evaluation: the IDEAL recommendations. The Lancet.

[8] Hirst A, Philippou Y, Blazeby J (2019). No Surgical Innovation Without Evaluation. Ann Surg.

[9] Pennell CP, Hirst AD, Campbell WB (2016). Practical guide to the Idea, Development and Exploration stages of the IDEAL Framework and Recommendations. Br J Surg.

[10] Bilbro NA (2020). New reporting guidelines for IDEAL studies. Br J Surg.

[11] Khachane A, Philippou Y, Hirst A (2018). Appraising the uptake and use of the IDEAL Framework and Recommendations: A review of the literature. Int J Surg.

[12] Tradewell MB, Albersheim J, Dahm P (2019). Use of the IDEAL framework in the urological literature: where are we in 2018?. BJU Int.

[13] Currie A, Brigic A, Blencowe NS (2015). Systematic review of surgical innovation reporting in laparoendoscopic colonic polyp resection. Br J Surg.

[14] Kirkham EN, Jones CS, Higginbotham G (2022). A systematic review of robot-assisted cholecystectomy to examine the quality of reporting in relation to the IDEAL recommendations: systematic review. BJS Open.

[15] Huttman MM, Robertson HF, Smith AN (2023). A systematic review of robot-assisted anti-reflux surgery to examine reporting standards. J Robot Surg.

[16] Hirst A, Philippou Y, Blazeby J (2019). No Surgical Innovation Without Evaluation: Evolution and Further Development of the IDEAL Framework and Recommendations. Ann Surg.

[17] Sedrakyan A, Campbell B, Merino JG (2016). IDEAL-D: a rational framework for evaluating and regulating the use of medical devices. BMJ.

[18] Marcus HJ, Bennett A, Chari A (2022). IDEAL-D Framework for Device Innovation: A Consensus Statement on the Preclinical Stage. Ann Surg.

[19] Hoffmann C, Hossaini S, Cousins S (2021). Reporting Modifications in Surgical Innovation: A Systematic Scoping Review Protocol. Int J Surg Protoc.

[20] Hossaini S, Hoffmann C, Cousins S (2023). Development of a conceptual framework for reporting modifications in surgical innovation: scoping review. BJS Open.

[21] Neugebauer EAM, Becker M, Buess GF (2010). EAES recommendations on methodology of innovation management in endoscopic surgery. Surg Endosc.

[22] Excellence NIfHaC (2016). Interventional procedures programme manual (PMG28).

[23] Hirst A, Agha RA, Rosin D (2013). How can we improve surgical research and innovation?: the IDEAL framework for action. Int J Surg.

[24] Hutchison K, Rogers W, Eyers A (2015). Getting Clearer About Surgical Innovation: A New Definition and a New Tool to Support Responsible Practice. Ann Surg.

[25] Rogers WA, Lotz M, Hutchison K (2014). Identifying surgical innovation: a qualitative study of surgeons’ views. Ann Surg.

[26] Blencowe NS, Brown JM, Cook JA (2015). Interventions in randomised controlled trials in surgery: issues to consider during trial design. Trials.

[27] Avery KNL, Wilson N, Macefield R (2023). A Core Outcome Set for Seamless, Standardized Evaluation of Innovative Surgical Procedures and Devices (COHESIVE): A Patient and Professional Stakeholder Consensus Study. Ann Surg.

[28] Macefield R, Scroggie D, Coyle M (2024). OP10 Insights into surgical innovation, incremental learning and refinement in practice: a case-study of aortic valve neocuspidization (avneo) with autologous pericardium (the ozaki procedure). BMJ Surgery Interventions, Health Technologies.

[29] Olivier J, Macefield R, Elliott D (2023). Identifying, classifying, reporting and evaluating modifications to surgical innovation: a systematic review of ideal/ideal-d studies: PROSPERO: international prospective register of systematic reviews. https://www.crd.york.ac.uk/prospero/display_record.php?ID=CRD42023427704.

[30] Page MJ, McKenzie JE, Bossuyt PM (2021). The PRISMA 2020 statement: an updated guideline for reporting systematic reviews. BMJ.

[31] Barkun JS, Aronson JK, Feldman LS (2009). Evaluation and stages of surgical innovations. The Lancet.

[32] Ergina PL, Cook JA, Blazeby JM (2009). Challenges in evaluating surgical innovation. The Lancet.

[33] Cook JA, McCulloch P, Blazeby JM (2013). IDEAL framework for surgical innovation 3: randomised controlled trials in the assessment stage and evaluations in the long term study stage. BMJ.

[34] Ergina PL, Barkun JS, McCulloch P (2013). IDEAL framework for surgical innovation 2: observational studies in the exploration and assessment stages. BMJ.

[35] McCulloch P, Cook JA, Altman DG (2013). IDEAL framework for surgical innovation 1: the idea and development stages. BMJ.

[36] Pennell CP, Hirst A, Sedrakyan A (2016). Adapting the IDEAL Framework and Recommendations for medical device evaluation: A modified Delphi survey. Int J Surg.

[37] Bilbro NA, Hirst A, Paez A (2021). The IDEAL Reporting Guidelines: A Delphi Consensus Statement Stage Specific Recommendations for Reporting the Evaluation of Surgical Innovation. Ann Surg.

[38] Falagas ME, Pitsouni EI, Malietzis GA (2008). Comparison of PubMed, Scopus, Web of Science, and Google Scholar: strengths and weaknesses. FASEB J.

[39] Linder SK, Kamath GR, Pratt GF (2015). Citation searches are more sensitive than keyword searches to identify studies using specific measurement instruments. J Clin Epidemiol.

[40] Macefield RC, Wilson N, Hoffmann C (2020). Outcome selection, measurement and reporting for new surgical procedures and devices: a systematic review of IDEAL/IDEAL-D studies to inform development of a core outcome set. BJS Open.

[41] The EndNote Team (2013). EndNote. EndNote 20 ed.

[42] Cousins S, Blencowe NS, Blazeby JM (2019). What is an invasive procedure? A definition to inform study design, evidence synthesis and research tracking. BMJ Open.

[43] Ouzzani M, Hammady H, Fedorowicz Z (2016). Rayyan-a web and mobile app for systematic reviews. Syst Rev.

[44] Higgins JPTTJ, Chandler J (2019). Cochrane database of systematic reviews.

[45] Harris PA, Taylor R, Minor BL (2019). The REDCap consortium: Building an international community of software platform partners. J Biomed Inform.

[46] Avery K, Blazeby J, Wilson N (2019). Development of reporting guidance and core outcome sets for seamless, standardised evaluation of innovative surgical procedures and devices: a study protocol for content generation and a Delphi consensus process (COHESIVE study). BMJ Open.

[47] Lumivero (2017). NVivo 12 pro [software].

[48] Booth A, Noyes J, Flemming K (2018). Structured methodology review identified seven (RETREAT) criteria for selecting qualitative evidence synthesis approaches. J Clin Epidemiol.

[49] Thomas J, Harden A (2008). Methods for the thematic synthesis of qualitative research in systematic reviews. BMC Med Res Methodol.

[50] Richards HS, Cousins S, Scroggie DL (2024). Examining the application of the IDEAL framework in the reporting and evaluation of innovative invasive procedures: secondary qualitative analysis of a systematic review. BMJ Open.

[51] McCulloch P, Feinberg J, Philippou Y (2018). Progress in clinical research in surgery and IDEAL. Lancet.

[52] Orczyk C, Barratt D, Brew-Graves C (2021). Prostate Radiofrequency Focal Ablation (ProRAFT) Trial: A Prospective Development Study Evaluating a Bipolar Radiofrequency Device to Treat Prostate Cancer. J Urol.

[53] Paleri V, Fox H, Coward S (2018). Transoral robotic surgery for residual and recurrent oropharyngeal cancers: Exploratory study of surgical innovation using the IDEAL framework for early‐phase surgical studies. Head Neck.

[54] Stenstra M, van Workum F, van den Wildenberg FJH (2019). Evolution of the surgical technique of minimally invasive Ivor-Lewis esophagectomy: description according to the IDEAL framework. Dis Esophagus.

[55] Diez del Val I, Loureiro C, McCulloch P (2015). The IDEAL prospective development study format for reporting surgical innovations. An illustrative case study of robotic oesophagectomy. Int J Surg.

[56] Blakely B, Selwood A, Rogers WA (2016). Macquarie Surgical Innovation Identification Tool (MSIIT): a study protocol for a usability and pilot test. BMJ Open.

