# Supplementary material for: Identifying and reporting modifications to surgical innovation: a systematic review of IDEAL/IDEAL-D studies
Source: BMJ Open. 2025 Jun 30;15(6):e097097. doi: 10.1136/bmjopen-2024-097097 (PMC12211834; doi:10.1136/bmjopen-2024-097097)
Supplement: online supplemental file 1 [file bmjopen-15-6-s001.docx]

**Supplementary file 1 – S1 – Search strategy**

**Search strategy:**

The search strategy for this study was prospectively registered on the PROSPERO database (CRD42023427704). It was based on a previous review conducted by Macefield et al in 2020, which examined outcome measurement and reporting in IDEAL-cited studies related to surgical innovation. This review identified studies that cited at least one of ten key IDEAL publications (see papers 1-10 below) using electronic citation tools in Web of Science and SCOPUS. Electronic citation searches in these databases were performed to identify studies reported as adhering to the IDEAL/IDEAL-D framework. This was defined as an article citing any of the ten key IDEAL/IDEAL-D papers that members of the IDEAL collaboration previously considered significant in describing the framework (see Khachane et al, 2018). Searches were conducted in April 2019.

This study also utilised this search strategy to identify IDEAL-citing studies on innovative surgical procedures and devices. Since the Khachane review, three additional key IDEAL/IDEAL-D papers have been published (see papers 11-13 below), resulting in a total of 13 key IDEAL papers. Searches were conducted in July 2023 for studies that cited at least one of these thirteen key IDEAL publications using the electronic citation tools in Web of Science (through Clarivate) and SCOPUS. The dates were restricted to January 2019 onwards to minimise duplication between the previous review by Macefield et al. and this review. There were no restrictions on study design or publication dates. Additionally, no study registries or other online or print sources were searched, and no further search methods were undertaken. Search filters were not required, given the nature of the citation searches. Due to the exploratory nature of the review, no further updates were deemed necessary following this date.

Web of Science

Search date: 5^th^ July 2023

901 records identified.

SCOPUS

Search date: 5th July 2023

983 records identified.

These records were combined with those from the previous 2020 review (see figure 1 – PRISMA flowchart) and uploaded to version 20 of EndNote, where de-duplication was performed. Records were then filtered to select those that included the words ‘IDEAL’ or ‘IDEAL-D’ in the title or abstract. This method for identifying and concentrating on IDEAL/IDEAL-D studies as the foundation for this review was based on the rationale that authors of studies aligned to the IDEAL framework are more likely to have considered the importance of reporting modifications, thereby providing a suitable data source for examining current practices. Study selection, data extraction, and analysis are detailed in the manuscript's main text.

**Citation searches were conducted for the following papers:**

1. McCulloch P, Altman DG, Campbell WB, Flum DR, Glasziou P, Marshall JC, et al. No surgical innovation without evaluation: the IDEAL recommendations. The Lancet. 2009;374(9695):1105-12.
2. Ergina PL, Cook JA, Blazeby JM, Boutron I, Clavien P-A, Reeves BC, et al. Challenges in evaluating surgical innovation. The Lancet. 2009;374(9695):1097-104.
3. Barkun JS, Aronson JK, Feldman LS, Maddern GJ, Strasberg SM. Evaluation and stages of surgical innovations. The Lancet. 2009;374(9695):1089-96.
4. Cook JA, McCulloch P, Blazeby JM, Beard DJ, Marinac-Dabic D, Sedrakyan A. IDEAL framework for surgical innovation 3: randomised controlled trials in the assessment stage and evaluations in the long term study stage. BMJ. 2013;346(jun18 3):f2820-f.
5. Ergina PL, Barkun JS, McCulloch P, Cook JA, Altman DG. IDEAL framework for surgical innovation 2: observational studies in the exploration and assessment stages. BMJ. 2013;346(jun18):f3011-f.
6. McCulloch P, Cook JA, Altman DG, Heneghan C, Diener MK. IDEAL framework for surgical innovation 1: the idea and development stages. BMJ. 2013;346(jun18 3):f3012-f.
7. Hirst A, Agha RA, Rosin D, McCulloch P. How can we improve surgical research and innovation?: The IDEAL framework for action. Int J Surg. 2013;11(10):1038-42.
8. Pennell CP, Hirst AD, Campbell WB, Sood A, Agha RA, Barkun JST, et al. Practical guide to the Idea, Development and Exploration stages of the IDEAL Framework and Recommendations. British Journal of Surgery. 2016;103(5):607-15.
9. Sedrakyan A, Campbell B, Merino JG, Kuntz R, Hirst A, McCulloch P. IDEAL-D: a rational framework for evaluating and regulating the use of medical devices. BMJ. 2016:i2372.
10. Pennell CP, Hirst A, Sedrakyan A, McCulloch PG. Adapting the IDEAL Framework and Recommendations for medical device evaluation: A modified Delphi survey. Int J Surg. 2016;28:141-8.
11. Hirst A, Philippou Y, Blazeby J, Campbell B, Campbell M, Feinberg J, et al. No Surgical Innovation Without Evaluation Evolution and Further Development of the IDEAL Framework and Recommendations. Ann Surg. 2019;269(2):211-20.
12. Bilbro NA, Hirst A, Paez A, Vasey B, Pufulete M, Sedrakyan A, et al. The IDEAL Reporting Guidelines A Delphi Consensus Statement Stage Specific Recommendations for Reporting the Evaluation of Surgical Innovation. Ann Surg. 2021;273(1):82-5.
13. Marcus HJ, Bennett A, Chari A, Day T, Hirst A, Hughes-Hallett A, et al. IDEAL-D Framework for Device Innovation A Consensus Statement on the Preclinical Stage. Ann Surg. 2022;275(1):73-9.
