# Supplementary material for: Identifying and reporting modifications to surgical innovation: a systematic review of IDEAL/IDEAL-D studies
Source: BMJ Open. 2025 Jun 30;15(6):e097097. doi: 10.1136/bmjopen-2024-097097 (PMC12211834; doi:10.1136/bmjopen-2024-097097)
Supplement: online supplemental file 2 [file bmjopen-15-6-s002.docx]

**Supplementary file 2 (S2) – Summary of Key characteristic of all 104 included papers for systematic review of IDEAL/IDEAL-D studies**

| **Authors** | **Journal** | **Year** | **Type of publication** | **Type of Innovation** | **Country/region of study** | **Number of centres** | **Number of participants** | **IDEAL stage(s)** | **Modification reporting** |
| --- | --- | --- | --- | --- | --- | --- | --- | --- | --- |
| Wang(1) | Chinese Journal of Cancer Research. | 2023 | study report | Procedure | China | 1 | 35 | 2a | Yes |
| Vogt(2) | The bone and joint journal | 2023 | study report | Device | EEA (European Economic Area) | 1 | 138 | 2a | Yes |
| Serra-Aracil(3) | Langenbeck's Archives of Surgery | 2023 | study report | Procedure | EEA | 1 | 79 | 2a | Yes |
| Moll(4) | International Journal of Hypothermia | 2023 | study report | Procedure | EEA | not reported | 8 | 2a | Yes |
| Klarenbeek (5) | BJS | 2023 | study report | Procedure | EEA | 1 | 3 | 0 + 1 | Yes |
| Ellebrecht (6) | Surgical Innovation | 2023 | study report | Device | EEA | 1 | 19 | 2a | No |
| Assmann (7) | Colorectal Disease | 2022 | study report | Device | EEA | 1 | 17 | 2a/2b | No |
| Veen (8) | BMC Cancer | 2022 | protocol | Procedure | EEA | 2 | 20 | 2a | Yes |
| Sood (9) | European Urology | 2022 | study report | Procedure | North America | 1 | 88 | 1, 2a/2b | Yes |
| Plaha (10) | BMJ Open | 2022 | protocol | Co-intervention | UK | 15 | 357 | 2b + 3 | No |
| Piana (11) | European Association of Urology | 2022 | study report | Co-intervention | EEA | not reported | 19 | 1 | Yes |
| Pausch (12) | BMJ Open | 2022 | protocol | Device | EEA | 1 | 35 | 1 | Yes |
| Nulens (13) | Journal of Obstetrics and Gynaecology | 2022 | study report | Procedure | EEA | 1 | 9 | 1 | Yes |
| Marra (14) | Frontiers in Surgery | 2022 | protocol | Procedure | Multiple | 1 | 20 | 2a | Yes |
| MacLeod (15) | Medical Engineering and Physics | 2022 | study report | Device | UK | 1 | 8 | 0 | No |
| lI (16) | Chinese Journal of Cancer Research | 2022 | protocol | Procedure | China | multi centre (not specified = NS) | 518 | 2b | No |
| Lenfant (17) | World Journal of Urology | 2022 | study report | Procedure | EEA | 1 | 2322 | 4 | Yes |
| Huber (18) | European Journal of Obstetrics and Gynecology and Reproductive Biology | 2022 | study report | Procedure | EEA | 1 | 15 | 2a | Yes |
| Harvey (19) | BJS | 2022 | study report | Procedure | UK | 40 | 347 | 2a/2b | Yes |
| Fleming (20) | BJS | 2022 | study report | Procedure | EEA | 1 | 35 | 2a | Yes |
| Dinneen (21) | Trials | 2022 | protocol | Procedure | UK | 4 | 404 | 3 | Yes |
| Maida (22) | European Journal of Surgical Oncology | 2022 | study report | Procedure | EEA | 1 | 100 | 3 | No |
| Dave (23) | BJS | 2022 | study report | Procedure | UK | 35 | 1003 | 2a/2b | Yes |
| Cabrilo (24) | Surgical Innovation | 2022 | study report | Device | UK | 1 | 5 | 1 | Yes |
| Brüggenwirth (25) | BMJ Open Gastroenterology | 2021 | protocol | Device | EEA | 1 | 18 | 2a | No |
| Borse (26) | Acta Obstet Gynecol Scand | 2022 | study report | Device | India | 3 | 144 | 2b | Yes |
| Vogt (27) | Bone and Joint Journal | 2021 | study report | Device | EEA | 1 | 56 | 2a | Yes |
| Vanhooren (28) | Asian J Endosc Surg | 2021 | study report | Procedure | EEA | 1 | 9 | 1 | Yes |
| Thomas (29) | European urology focus | 2021 | study report | Device | UK | 1 | 24 | 0 | Yes |
| Territo (30) | European Association of Urology. | 2021 | study report | Device | Multiple | 1 | 5 | 0 + 1 | Yes |
| Robertson (31) | BJS open | 2021 | study report | Procedure | Canada | 1 | 114 | 2a/2b (Reviewer allocated = RA) | Yes |
| Orczyk (32) | The Journal of Urology | 2021 | study report | Device | UK | not reported | 20 | 2a | Yes |
| Paleri (33) | Head and neck | 2018 | study report | Procedure | UK | 1 | 21 | 2a | Yes |
| Minervini (34) | European Journal of Surgical Oncology | 2021 | study report | Procedure | EEA | 1 | 100 | 3 | Yes |
| Mehrabi (35) | Mehrabi | 2021 | protocol | Procedure | UK | 1 | 20 | 2a | No |
| Kelkar (36) | Surgical Endoscopy | 2021 | study report | Device | India | 1 | 30 | 2a | Yes |
| Kaouk (37) | European Urology | 2021 | study report | Procedure | North America | 1 | 9 | 1 | Yes |
| Collins (38) | Colorectal Disease | 2021 | study report | Device | UK | 1 | 32 | 2a | Yes |
| Chen (39) | Journal of Orthopaedic Translation | 2021 | study report | Device | Multiple | 2 | 18 | 1 + 2a | No |
| Ahlawat (40) | Journal of Urology | 2021 | study report | Procedure | India | 1 | 126 | 2b | Yes |
| Wishahi (41) | Central European Journal of Urology | 2020 | study report | Procedure | Egypt | 1 | 56 | 2a | Yes |
| Kollenburg (42) | JMIR Research Protocols | 2020 | protocol | Device | EEA | 1 | 20 | 2a | No |
| Shen (43) | Medicine (United States) | 2020 | protocol | Procedure | China | multi centre (NS) | 30 | 2a | Yes |
| Schilling (44) | European Journal of Surgical Oncology | 2020 | study report | Procedure | UK | multi centre (NS) | 10 | 2a (RA) | Yes |
| Rassweiler-Seyfried (45) | World Journal of Urology | 2020 | study report | Procedure | EEA | 1 | 22 | 2b | No |
| Pausch (46) | Journal of Biomaterials | 2020 | study report | Device | EEA | 1 | 20 | 0 (RA) | Yes |
| Koumoullis (47) | British Journal of Oral and Maxillofacial Surgery | 2020 | study report | Procedure | UK | 1 | 1 | 1 | No |
| Harvey (48) | Bmj Open | 2020 | protocol | Procedure | UK | 20-30 | 341 | 2a/2b | Yes |
| Dumont (49) | Surgical Endoscopy | 2020 | study report | Procedure | EEA | not reported | 12 | 1/2a | Yes |
| Basourakos (50) | BMJ Surgery, Interventions, and Health Technologies | 2020 | study report | Procedure | North America | 2 | 55 | 2a | Yes |
| Tzeng (51) | BMJ Surgery | 2019 | study report | Procedure | North America | not reported | 33 | 2a | Yes |
| Stenstra (52) | Diseases of the Esophagus | 2019 | study report | Procedure | EEA | 1 | 164 | 2b + 3 | Yes |
| Sood (53) | BMJ Surgery | 2019 | study report | Procedure | North America | 1 | 108 | 0, 1 + 2a | Yes |
| Sharma (54) | International Journal of Surgery | 2019 | study report | Device | North America | 1 | 30 | 1 | Yes |
| Gaboardi (55) | Journal of Robotic Surgery | 2019 | study report | Procedure | EEA | 1 | 12 | 1 | Yes |
| Elsanousi (56) | International Journal of Surgery | 2019 | study report | Procedure | Sudan | 1 | 20 | 2a | Yes |
| Rooij (57) | Annals of surgery | 2019 | study report | Procedure | Multiple | 14 | 51 | 3 (RA) | Yes |
| Currie (58) | Colorectal Disease | 2019 | study report | Procedure | UK | 1 | 10 | 2a (RA) | Yes |
| Chan (59) | Oral Oncology | 2019 | study report | Device | Multiple | not reported | 21 | 2a | Yes |
| Versteeg (60) | Frontiers in Oncology | 2018 | study report | Procedure | EEA | 1 | 13 | 1/2a | Yes |
| Swaan (61) | Jmir Research Protocols | 2018 | protocol | Procedure | Multiple | 2 | 14 | 2a | No |
| Springer (62) | Urology | 2018 | study report | Procedure | EEA | 1 | 40 | 2a | No |
| Minervini (63) | Bju International | 2018 | study report | Procedure | EEA | 1 | 18 | 1, 2a + 2b | Yes |
| Mannaerts (64) | Bmc Urology | 2018 | protocol | Co-intervention | Multiple | 1 | 50 | 2b | No |
| Lim (65) | International urogynecology journal | 2018 | study report | Procedure | Malaysia | multi centre (NS) | 60 | 2b | No |
| Liem (66) | Jmir Research Protocols | 2018 | protocol | Device | EEA | 1 | 85 | 2b | Yes |
| Kidane (67) | Surgical Infections | 2018 | study report | Procedure | Canada | 1 | 19 | 2a/2b | Yes |
| Graff (68) | International Journal of Radiation Oncology Biology Physics | 2018 | study report | Procedure | EEA | 1 | 17 | 2a | No |
| Chen (69) | Bjog-an International Journal of Obstetrics and Gynaecology | 2018 | study report | Procedure | China | 20 | 1353 | 2b | No |
| Chandak (70) | Bju International | 2018 | study report | Co-intervention | UK | 1 | 10 | 2a | No |
| Breda (71) | European Urology Focus | 2018 | study report | Co-intervention | EEA | 1 | 12 | 2a (RA) | No |
| Banagala (72) | Bmc Surgery | 2018 | study report | Procedure | Sri Lanka | 1 | 32 | 2a | Yes |
| Villers (73) | European Urology | 2017 | study report | Procedure | EEA | 1 | 17 | 2a | Yes |
| Rischmann (74) | European Urology | 2017 | study report | Procedure | EEA | 10 | 111 | 2b | No |
| Hallmann (75) | Clinical Case Reports | 2017 | study report | Procedure | EEA | 1 | 1 | 1 | No |
| Gerullis (76) | Urologia Internationalis | 2017 | study report | Procedure | EEA | 1 | 14 | 2a | Yes |
| Gerullis (77) | Journal of International Medical Research | 2017 | study report | Procedure | EEA | 1 | 3 | 1 | Yes |
| Gerullis (78) | Advances in Therapy | 2017 | protocol | Procedure | EEA | 2 | 252 | 3 | Yes |
| Ecke (79) | International Journal of Medical Sciences | 2017 | study report | Procedure | EEA | 1 | 226 | 2b | No |
| Douglas (80) | JPRAS Open | 2017 | study report | Procedure | UK | 1 | 1 | 1 | Yes |
| Buijs (81) | Jmir Research Protocols | 2017 | protocol | Procedure | EEA | 1 | 20 | 2b | No |
| Barski (82) | International Journal of Surgery | 2017 | study report | Co-intervention | EEA | 1 | 20 | Unable to stage | Yes |
| Barski (83) | International Journal of Surgery | 2017 | study report | Device | EEA | 1 | 34 | 1 | Yes |
| Baekelandt (84) | BMJ innovations | 2017 | study report | Device | EEA | 1 | 10 | 1 | Yes |
| Hoven (85) | Cardiovascular and Interventional Radiology | 2016 | study report | Co-intervention | EEA | 1 | 50 | 2a | Yes |
| Sood (86) | International Journal of Surgery | 2016 | study report | Procedure | Multiple | not reported | 54 | 0, 1 + 2a | Yes |
| Joukhadar (87) | BioMed Research International | 2016 | study report | Procedure | EEA | 1 | 129 | 2b | Yes |
| Inoue (88) | Langenbecks Archives of Surgery | 2016 | study report | Procedure | Japan | 1 | 5 | 1 | Yes |
| Greco (89) | European Urology Focus | 2016 | study report | Procedure | EEA | 1 | 20 | 2a | No |
| Bus (90) | Journal of Urology | 2016 | study report | Procedure | EEA | 1 | 26 | 2a + 2b | No |
| Kroeze (91) | World Journal of Urology | 2015 | study report | Procedure | EEA | 1 | 10 | 2a | Yes |
| Diez del Val (92) | International Journal of Surgery | 2015 | study report | Procedure | EEA | 1 | 35 | 2a | Yes |
| Barski (93) | Central European Journal of Urology | 2015 | study report | Procedure | EEA | 1 | 1 | 1 | No |
| Barentsz (94) | International Journal of Surgery | 2015 | study report | Procedure | EEA | 1 | 12 | 2a | Yes |
| Valerio (95) | Contemporary Clinical Trials | 2014 | protocol | Procedure | UK | 1 | 20 | 2a | Yes |
| Sood (96) | European Urology | 2014 | study report | Procedure | Multiple | 2 | 41 | 2a/2b | Yes |
| Saglam (97) | European Urology | 2014 | study report | Device | Multiple | not reported | 81 | 1, 2a + 2b | Yes |
| Kaouk (98) | European Urology | 2014 | study report | Device | Multiple | 1 | 22 | 1 | Yes |
| Menon (99) | European Urology | 2014 | study report | Procedure | India | 1 | 7 | 0 + 1 | Yes |
| Heikens (100) | Surgical Innovation | 2013 | study report | Procedure | EEA | 2 | 58 | 0, 1 , 2a, 2b + 3 | Yes |
| Kroeze (101) | Cardiovascular and Interventional Radiology | 2012 | study report | Co-intervention | EEA | 1 | 13 | 2a | No |
| Solari (102) | Journal of Pediatric Surgery | 2011 | study report | Procedure | North America | 1 | 1 | 1 (RA) | Yes |
| Diepstraten (103) | Cancer Imaging | 2011 | study report | Co-intervention | EEA | 1 | 19 | 2a (RA) | Yes |
| Blazeby (104) | British Journal of Surgery | 2011 | study report | Procedure | UK | 1 | 124 | 1, 2a + 2b | Yes |

**References**

1. Wang YK, Hong FL, Li SX, Shan F, Jia YN, Miao RL, et al. Exploration and optimization of surgical techniques for laparoscopic transhiatal lower mediastinal lymph node dissection for adenocarcinoma of esophagogastric junction: A prospective IDEAL 2a study with qualitative design. Chinese Journal of Cancer Research. 2023;35(2):163-+.

2. Vogt B, Toporowski G, Gosheger G, Laufer A, Frommer A, Kleine-Koenig MT, et al. Guided growth: angular deformity correction through temporary hemiepiphysiodesis with a novel flexible staple (FlexTack). Bone and Joint Journal. 2023;105(3):331-40.

3. Serra-Aracil X, Mora-Lopez L, Gomez-Torres I, Pallisera-Lloveras A, Serracant A, Garcia-Nalda A, et al. Laparoscopic and robotic intracorporeal resection and end-to-end anastomosis in left colectomy: a prospective cohort study — stage 2a IDEAL framework for evaluating surgical innovation. Langenbeck's Archives of Surgery. 2023;408(1).

4. Moll X, Fondevila D, García-Arnas F, Pérez JJ, Ielpo B, Sánchez-Velázquez P, et al. Is occlusion of the main pancreatic duct by thermal ablation really safe? A surgical innovation assessed according to IDEAL recommendations. International Journal of Hyperthermia. 2023;40(1).

5. Klarenbeek BR, Fujiwara H, Scholte M, Rovers M, Shiozaki A, Rosman C. Introduction of Minimally Invasive transCervical oEsophagectomy (MICE) according to the IDEAL framework. British Journal of Surgery. 2023:4.

6. Ellebrecht DB, Kugler C. Intraoperative Determination of Bronchus Stump and Anastomosis Perfusion with Hyperspectral Imaging. Surgical Innovation. 2023.

7. Assmann R, Douven P, Joosten EA, van Koeveringe GA, Breukink SO, Melenhorst J. Replacement Adaptor 09106 for patients with a dynamic graciloplasty or patients with sacral neuromodulation and abdominal implantable pulse generators: a retrospective, single centre, Stage 2a/2b development IDEAL case series. Colorectal Disease. 2023;25(1):150-5.

8. Veen A, Schiffmann LM, de Groot EM, Bartella I, de Jong PA, Borggreve AS, et al. The ISCON-trial protocol: laparoscopic ischemic conditioning prior to esophagectomy in patients with esophageal cancer and arterial calcifications. BMC Cancer. 2022;22(1).

9. Sood A, Jeong W, Palma-Zamora I, Abdollah F, Butaney M, Corsi N, et al. Description of Surgical Technique and Oncologic and Functional Outcomes of the Precision Prostatectomy Procedure (IDEAL Stage 1–2b Study). European Urology. 2022;81(4):396-406.

10. Plaha P, Camp S, Cook J, McCulloch P, Voets N, Ma R, et al. FUTURE-GB: functional and ultrasound-guided resection of glioblastoma - a two-stage randomised control trial. BMJ Open. 2022;12(11).

11. Piana A, Gallioli A, Amparore D, Diana P, Territo A, Campi R, et al. Three-dimensional Augmented Reality–guided Robotic-assisted Kidney Transplantation: Breaking the Limit of Atheromatic Plaques. European Urology. 2022;82(4):419-26.

12. Pausch TM, Holze M, Gesslein B, Rossion I, Von Eisenhart Rothe F, Wagner M, et al. Intraoperative visualisation of pancreatic leakage (ViP): study protocol for an IDEAL Stage I Post Market Clinical Study. BMJ Open. 2022;12(9).

13. Nulens K, Kempenaers R, Baekel, t J. Hysterectomy via vaginal Natural Orifice Transluminal Endoscopic Surgery in virgin patients: a first feasibility study. Journal of Obstetrics and Gynaecology. 2022;42(1):116-21.

14. Marra G, Shah TT, D’Agate D, Marquis A, Calleris G, Lunelli L, et al. The SAFE Pilot Trial—SAlvage Focal Irreversible Electroporation—For Recurrent Localized Prostate Cancer: Rationale and Study Protocol. Frontiers in Surgery. 2022;9.

15. MacLeod AR, alia VI, Mathews JA, Toms AD, Gill HS. Personalised 3D Printed high tibial osteotomy achieves a high level of accuracy: ‘IDEAL’ preclinical stage evaluation of a novel patient specific system. Medical Engineering and Physics. 2022;108.

16. Li SX, Ying XJ, Shan F, Jia YN, Li ZM, Xue K, et al. Laparoscopic vs. open lower mediastinal lymphadenectomy for Siewert type II/III adenocarcinoma of esophagogastric junction: An exploratory, observational, prospective, IDEAL stage 2b cohort study (CLASS-10 study). Chinese Journal of Cancer Research. 2022;34(4):406-+.

17. Lenfant L, Renard-Penna R, de Rycke Y, Rouprêt M, Beaugerie A, Comperat E, et al. Dynamic evaluation of MRI-targeted, systematic and combined biopsy for prostate cancer diagnosis through 10 years of practice in a single institution. World Journal of Urology. 2022;40(7):1661-8.

18. Huber D, Fournier I, Christodoulou M, Seidler S, Besse V, Mathey MP, et al. Stapled diaphragm resection: A new approach to diaphragmatic cytoreductive surgery for advanced-stage ovarian cancer. European Journal of Obstetrics and Gynecology and Reproductive Biology. 2022;279:88-93.

19. Harvey KL, Sinai P, Mills N, White P, Holcombe C, Potter S, et al. Short-Term safety outcomes of mastectomy and immediate prepectoral implant-based breast reconstruction: Pre-BRA prospective multicentre cohort study. British Journal of Surgery. 2022;109(6):530-8.

20. Fleming CA, Harji D, Salut C, Cauvin T, Robert G, Denost Q. Robotic-assisted soft-tissue pelvic exenteration for primary and recurrent pelvic tumours: IDEAL stage 2a evaluation. British Journal of Surgery. 2022:4.

21. Dinneen E, Grierson J, Almeida-Magana R, Clow R, Haider A, Allen C, et al. NeuroSAFE PROOF: study protocol for a single-blinded, IDEAL stage 3, multi-centre, randomised controlled trial of NeuroSAFE robotic-assisted radical prostatectomy versus standard robotic-assisted radical prostatectomy in men with localized prostate cancer. Trials. 2022;23(1).

22. Di Maida F, Grosso AA, Tasso G, Gemma L, Lambertini L, Nardoni S, et al. Robot assisted radical cystectomy with Florence Robotic Intracorporeal Neobladder (FloRIN): Functional and urodynamic features compared with a contemporary series of open Vescica Ileale Padovana (VIP). European Journal of Surgical Oncology. 2022;48(8):1854-61.

23. Dave RV, Barrett E, Morgan J, Ch, arana M, Elgammal S, et al. Wire-and magnetic-seed-guided localization of impalpable breast lesions: IBRA-NET localisation study. British Journal of Surgery. 2022;109(3):274-82.

24. Cabrilo I, Delaunay R, Heaysman CL, Ourselin S, Vitiello V, Vercauteren T, et al. A Novel Intraoperative Ultrasound Probe for Transsphenoidal Surgery: First-in-human study. Surgical Innovation. 2022;29(2):282-8.

25. Brüggenwirth IMA, Lantinga VA, Rayar M, Van Den Berg AP, Blokzijl H, Reyntjens KMEM, et al. Prolonged dual hypothermic oxygenated machine preservation (DHOPE-PRO) in liver transplantation: Study protocol for a stage 2, prospective, dual-arm, safety and feasibility clinical trial. BMJ Open Gastroenterology. 2022;9(1).

26. Borse M, Godbole G, Kelkar D, Bahulikar M, Dinneen E, Slack M. Early evaluation of a next-generation surgical system in robot-assisted total laparoscopic hysterectomy: A prospective clinical cohort study. Acta Obstetricia et Gynecologica Scandinavica. 2022;101(9):978-86.

27. Vogt B, Roedl R, Gosheger G, Frommer A, Laufer A, Kleine-Koenig MT, et al. Growth arrest: Leg length correction through temporary epiphysiodesis with a novel rigid staple (RigidTack). Bone and Joint Journal. 2021;103(8):1428-37.

28. Vanhooren E, Baekel, t J. Vaginal NOTES surgery in patients with prior hysterectomy: A first case series. Asian Journal of Endoscopic Surgery. 2021;14(4):811-5.

29. Thomas BC, Slack M, Hussain M, Barber N, Pradhan A, Dinneen E, et al. Preclinical Evaluation of the Versius Surgical System, a New Robot-assisted Surgical Device for Use in Minimal Access Renal and Prostate Surgery. European Urology Focus. 2021;7(2):444-52.

30. Territo A, Piana A, Fontana M, Diana P, Gallioli A, Gaya JM, et al. Step-by-step Development of a Cold Ischemia Device for Open and Robotic-assisted Renal Transplantation. European Urology. 2021;80(6):738-45.

31. Robertson RL, Karimuddin A, Phang T, Raval M, Brown C. Transanal versus conventional total mesorectal excision for rectal cancer using the IDEAL framework for implementation. BJS Open. 2021;5(2).

32. Orczyk C, Barratt D, Brew-Graves C, Hu YP, Freeman A, McCartan N, et al. Prostate Radiofrequency Focal Ablation (ProRAFT) Trial: A Prospective Development Study Evaluating a Bipolar Radiofrequency Device to Treat Prostate Cancer. JOURNAL OF UROLOGY. 2021;205(4):1090-8.

33. Paleri V, Fox H, Coward S, Ragbir M, McQueen A, Ahmed O, et al. Transoral robotic surgery for residual and recurrent oropharyngeal cancers: Exploratory study of surgical innovation using the IDEAL framework for early-phase surgical studies. Head and Neck-Journal for the Sciences and Specialties of the Head and Neck. 2018;40(3):512-25.

34. Minervini A, Di Maida F, Tasso G, Mari A, Bossa R, Sforza S, et al. Robot assisted radical cystectomy with Florence robotic intracorporeal neobladder (FloRIN): Analysis of survival and functional outcomes after first 100 consecutive patients upon accomplishment of phase 3 IDEAL framework. European Journal of Surgical Oncology. 2021;47(10):2651-7.

35. Mehrabi A, Loos M, Ramouz A, Dooghaie Moghadam A, Probst P, Nickel F, et al. Gastric venous reconstruction to reduce gastric venous congestion after total pancreatectomy: Study protocol of a single-centre prospective non-randomised observational study (IDEAL Phase 2A) - GENDER study (Gastric v en ous D rainag e R econstruction). BMJ Open. 2021;11(10).

36. Kelkar D, Borse MA, Godbole GP, Kurlekar U, Slack M. Interim safety analysis of the first-in-human clinical trial of the Versius surgical system, a new robot-assisted device for use in minimal access surgery. Surgical Endoscopy. 2021;35(9):5193-202.

37. Kaouk J, Eltemamy M, Aminsharifi A, Schwen Z, Wilson C, Abou Zeinab M, et al. Initial Experience with Single-port Robotic-assisted Kidney Transplantation and Autotransplantation. European Urology. 2021;80(3):366-73.

38. Collins D, Paterson HM, Skipworth RJE, Speake D. Implementation of the Versius robotic surgical system for colorectal cancer surgery: First clinical experience. Colorectal Disease. 2021;23(5):1233-8.

39. Chen P, Wang A, Haynes W, ao-Bassonga E, Lee C, Ruan R, et al. A bio-inductive collagen scaffold that supports human primary tendon-derived cell growth for rotator cuff repair. Journal of Orthopaedic Translation. 2021;31:91-101.

40. Ahlawat R, Sood A, Jeong W, Ghosh P, Keeley J, Abdollah F, et al. Robotic Kidney Transplantation with Regional Hypothermia versus Open Kidney Transplantation for Patients with End Stage Renal Disease: An Ideal Stage 2B Study. Journal of Urology. 2021;205(2):595-602.

41. Wishahi M, Elkholy A, Badawy MH. Repair of distal hypospadias by construction of neourethra from augmented urethral plate with two lateral strips of glans skin and coverage with dartos flap followed by skin closure with preputial flap: Single center series. Central European Journal of Urology. 2020;73(4):1-8.

42. van Kollenburg RAA, van Riel LAMJG, Bloemen PR, Oddens JR, de Reijke TM, Beerlage HP, et al. Transperineal laser ablation treatment for lower urinary tract symptoms due to benign prostatic obstruction: Protocol for a prospective in vivo pilot study. JMIR Research Protocols. 2020;9(1).

43. Shen Y, Yang T, Deng X, Yang J, Meng W, Wang Z. Pelvic peritoneum reconstruction using the bladder peritoneum flap in laparoscopic extralevator abdominoperineal excision: A multi-center, prospective single-arm cohort study (IDEAL Phase 2A). Medicine (United States). 2020;99(25):E20712.

44. Schilling C, Gnanasegaran G, Thavaraj S, Vojnovic B, Ngu R, McGurk M. Development of sentinel lymph node biopsy technique in patients with salivary gland cancer using the IDEAL framework. European Journal of Surgical Oncology. 2020;46(11):2029-34.

45. Rassweiler-Seyfried MC, Rassweiler JJ, Weiss C, Müller M, Meinzer HP, Maier-Hein L, et al. iPad-assisted percutaneous nephrolithotomy (PCNL): a matched pair analysis compared to standard PCNL. World Journal of Urology. 2020;38(2):447-53.

46. Pausch TM, Mitzscherling C, Abbasi S, Cui J, Liu X, Aubert O, et al. SmartPAN: A novel polysaccharide-microsphere-based surgical indicator of pancreatic leakage. Journal of Biomaterials Applications. 2020;35(1):123-34.

47. Koumoullis H, Burley O, Kyzas P. Patient-specific soft tissue reconstruction: an IDEAL stage I report of hemiglossectomy reconstruction and introduction of the PANSOFOS flap. British Journal of Oral and Maxillofacial Surgery. 2020;58(6):681-6.

48. Harvey KL, Mills N, White P, Holcombe C, Potter S, Pre BRAFSS. The Pre-BRA (pre-pectoral Breast Reconstruction EvAluation) feasibility study: protocol for a mixed-methods IDEAL 2a/2b prospective cohort study to determine the safety and effectiveness of prepectoral implant-based breast reconstruction. Bmj Open. 2020;10(1):7.

49. Dumont F, Duchalais E, Aumont A, Thibaudeau E. Cytoreductive surgery plus hyperthermic intraperitoneal chemotherapy by laparoscopy via a single-port approach for low-grade peritoneal malignancy. Surgical Endoscopy. 2020;34(6):2789-95.

50. Basourakos SP, Al Hussein Al Awamlh B, Bianco FJ, Patel NA, Laviana A, Margolis DJ, et al. Feasibility of in-office MRI-targeted partial gland cryoablation for prostate cancer: An IDEAL stage 2A study. BMJ Surgery, Interventions, and Health Technologies. 2020;2(1).

51. Tzeng M, Cricco-Lizza E, Al Hussein Al Awamlh B, Pantuck M, Margolis DJ, Yu M, et al. IDEAL Stage 2a experience with in-office, transperineal MRI/ultrasound software fusion targeted prostate biopsy. BMJ Surgery, Interventions, and Health Technologies. 2019;1(1).

52. Stenstra M, van Workum F, van den Wildenberg FJH, Polat F, Rosman C. Evolution of the surgical technique of minimally invasive Ivor-Lewis esophagectomy: description according to the IDEAL framework. Diseases of the Esophagus. 2019;32(3):8.

53. Sood A, Jeong W, Taneja K, Abdollah F, Palma-Zamora I, Arora S, et al. The Precision Prostatectomy: An IDEAL Stage 0, 1 and 2a Study. BMJ Surgery, Interventions, and Health Technologies. 2019;1(1).

54. Sharma SK, Momose K, Sedrakyan A, Sonoda T, Sharaiha RZ. Endoscopic stabilization device evaluation using IDEAL framework: A quality improvement study. Int J Surg. 2019;67:18-23.

55. Gaboardi F, Pini G, Suardi N, Montorsi F, Passaretti G, Smelzo S. Robotic laparoendoscopic single-site radical prostatectomy (R-LESS-RP) with daVinci Single-Site® platform. Concept and evolution of the technique following an IDEAL phase 1. Journal of Robotic Surgery. 2019;13(2):215-26.

56. Elsanousi OM, Mohamed MA, Salim FH, Adam EA. Selective devascularization treatment for large hepatocellular carcinoma: Stage 2A IDEAL prospective case series. Int J Surg. 2019;68:134-41.

57. De Rooij T, Van Hilst J, Van Santvoort H, Boerma D, Van Den Boezem P, Daams F, et al. Minimally Invasive Versus Open Distal Pancreatectomy (LEOPARD): A Multicenter Patient-blinded Randomized Controlled Trial. Ann Surg. 2019;269(1):2-9.

58. Currie AC, Blazeby JM, Suzuki N, Thomas-Gibson S, Reeves B, Morton D, et al. Evaluation of an early-stage innovation for full-thickness excision of benign colonic polyps using the IDEAL framework. Colorectal Disease. 2019;21(9):1004-16.

59. Chan JYK, Tsang RK, Holsinger FC, Tong MCF, Ng CWK, Chiu PWY, et al. Prospective clinical trial to evaluate safety and feasibility of using a single port flexible robotic system for transoral head and neck surgery. Oral Oncology. 2019;94:101-5.

60. Versteeg AL, van der Velden JM, Hes J, Eppinga W, Kasperts N, Verkooijen HM, et al. Stereotactic Radiotherapy Followed by Surgical Stabilization Within 24 h for Unstable Spinal Metastases; A Stage I/IIa Study According to the IDEAL Framework. Frontiers in Oncology. 2018;8:7.

61. Swaan A, Mannaerts CK, Scheltema MJV, Nieuwenhuijzen JA, Savci-Heijink CD, de la Rosette J, et al. Confocal Laser Endomicroscopy and Optical Coherence Tomography for the Diagnosis of Prostate Cancer: A Needle-Based, In Vivo Feasibility Study Protocol (IDEAL Phase 2A). Jmir Research Protocols. 2018;7(5):11.

62. Springer C, Kawan F, La Rocca R, Mohammed N, Fornara P, Mirone V, et al. New Hybrid Mini-laparoendoscopic Single-site Partial Nephrectomy With Early Unclamped Technique for Renal Tumors With Intermediate PADUA Score (IDEAL Phase 2a). Urology. 2018;111:104-9.

63. Minervini A, Vanacore D, Vittori G, Milanesi M, Tuccio A, Siena G, et al. Florence robotic intracorporeal neobladder (FloRIN): a new reconfiguration strategy developed following the IDEAL guidelines. Bju International. 2018;121(2):313-7.

64. Mannaerts CK, Wildeboer RR, Postema AW, Hagemann J, Budaus L, Tilki D, et al. Multiparametric ultrasound: evaluation of greyscale, shear wave elastography and contrast-enhanced ultrasound for prostate cancer detection and localization in correlation to radical prostatectomy specimens. Bmc Urology. 2018;18:10.

65. Lim R, Liong ML, Leong WS, Khan NAK, Yuen KH. Effect of pulsed magnetic stimulation on quality of life of female patients with stress urinary incontinence: an IDEAL-D stage 2b study. International Urogynecology Journal. 2018;29(4):547-54.

66. Liem E, Freund JE, Baard J, de Bruin DM, Pes MPL, Savci-Heijink CD, et al. Confocal Laser Endomicroscopy for the Diagnosis of Urothelial Carcinoma in the Bladder and the Upper Urinary Tract: Protocols for Two Prospective Explorative Studies. Jmir Research Protocols. 2018;7(2):9.

67. Kidane B, Lung K, McCreery G, El-Khatib C, Ott MC, Hernandez-Alejandro R, et al. Early Rescue from Acute Severe Clostridium Difficile: A Novel Treatment Strategy. Surg Infect. 2018;19(1):78-82.

68. Graff P, Portalez D, Lusque A, Brun T, Aziza R, Khalifa J, et al. IDEAL 2a Phase II Study of Ultrafocal Brachytherapy for Low- and Intermediate-risk Prostate Cancer. International Journal of Radiation Oncology Biology Physics. 2018;102(4):903-11.

69. Chen J, Li Y, Wang Z, McCulloch P, Hu L, Chen W, et al. Evaluation of high-intensity focused ultrasound ablation for uterine fibroids: an IDEAL prospective exploration study. Bjog-an International Journal of Obstetrics and Gynaecology. 2018;125(3):354-64.

70. Chandak P, Byrne N, Lynch H, Allen C, Rottenberg G, Chandra A, et al. Three-dimensional printing in robot-assisted radical prostatectomy - an Idea, Development, Exploration, Assessment, Long-term follow-up (IDEAL) Phase 2a study. Bju International. 2018;122(3):360-1.

71. Breda A, Territo A, Guttilla A, Sanguedolce F, Manfredi M, Quaresima L, et al. Correlation Between Confocal Laser Endomicroscopy (Cellvizio (R)) and Histological Grading of Upper Tract Urothelial Carcinoma: A Step Forward for a Better Selection of Patients Suitable for Conservative Management. European Urology Focus. 2018;4(6):954-9.

72. Banagala S, Jayarajah U, Almeida I, Samarasekera DN. Efficacy of irrigation tubes in the management of para rectal cavities associated with complex fistula-in-ano. Bmc Surgery. 2018;18:6.

73. Villers A, Puech P, Flamand V, Haber GP, Desai MM, Crouzet S, et al. Partial Prostatectomy for Anterior Cancer: Short-term Oncologic and Functional Outcomes. European Urology. 2017;72(3):333-42.

74. Rischmann P, Gelet A, Riche B, Villers A, Pasticier G, Bondil P, et al. Focal High Intensity Focused Ultrasound of Unilateral Localized Prostate Cancer: A Prospective Multicentric Hemiablation Study of 111 Patients. European Urology. 2017;71(2):267-73.

75. Hallmann S, Petersein J, Ruttloff J, Ecke TH. Successful evacuation of large perirenal hematoma after extracorporeal shock wave lithotripsy (ESWL) - step 1 of the IDEAL recommendations of surgical innovation. Clinical Case Reports. 2017;5(2):123-5.

76. Gerullis H, Schwartmann K, Ecke TH, Georgas E, Herberg P, Winter A, et al. Transvesical Suprapubic Externalization of Ureteral Stents - Introduction of a Surgical Innovation at the Development Stage. Urologia Internationalis. 2017;99(1):69-76.

77. Gerullis H, Ecke TH, Barski D, Bantel C, Weyland A, Uphoff J, et al. Retrospective analysis of a surgical innovation using the IDEAL framework: radical cystectomy with epidural anaesthesia. Journal of International Medical Research. 2017;45(2):714-22.

78. Gerullis H, Barski D, Georgas E, Boros M, Ramon A, Ecke TH, et al. Protocol for a Randomized Phase II Trial for Mesh Optimization by Autologous Plasma Coating in Prolapse Repair: IDEAL Stage 3. Advances in Therapy. 2017;34(4):995-1006.

79. Ecke TH, Barski D, Weingart G, Lange C, Hallmann S, Ruttloff J, et al. Presentation of a method at the Exploration Stage according to IDEAL: Percutaneous nephrolithotomy (PCNL) under local infiltrative anesthesia is a feasible and effective method - retrospective analysis of 439 patients. International Journal of Medical Sciences. 2017;14(4):302-9.

80. Douglas AJW, Kyzas PA. A new autologous block-bone prefabricated flap concept based on the supraclavicular artery island flap (SCAIF) for reconstruction of a neo-mandibular osteoradionecrosis (ORN) defect, IDEAL Stage 1 report. JPRAS Open. 2017;12:19-24.

81. Buijs M, van Lienden KP, Wagstaff PGK, Scheltema MJV, de Bruin DM, Zondervan PJ, et al. Irreversible Electroporation for the Ablation of Renal Cell Carcinoma: A Prospective, Human, In Vivo Study Protocol (IDEAL Phase 2b). Jmir Research Protocols. 2017;6(2):12.

82. Barski D, Gerullis H, Ecke T, Kranz J, Schneidewind L, Leistner N, et al. Registry of implants for the reconstruction of pelvic floor in males and females: A feasibility case series. Int J Surg. 2017;42:27-33.

83. Barski D, Arndt C, Gerullis H, Yang J, Boros M, Otto T, et al. Transvaginal PVDF-mesh for cystocele repair: A cohort study. Int J Surg. 2017;39:249-54.

84. Baekelandt J, Bosteels J. Hysterectomy through the looking glass: iHysterectomy frugal by iPhone. BMJ Innovations. 2017;3(2):71-5.

85. van den Hoven AF, Prince JF, de Keizer B, Vonken E, Bruijnen RCG, Verkooijen HM, et al. Use of C-Arm Cone Beam CT During Hepatic Radioembolization: Protocol Optimization for Extrahepatic Shunting and Parenchymal Enhancement. Cardiovascular and Interventional Radiology. 2016;39(1):64-73.

86. Sood A, McCulloch P, Dahm P, Ahlawat R, Jeong WJ, Bhandari M, et al. Ontogeny of a surgical technique: Robotic kidney transplantation with regional hypothermia. Int J Surg. 2016;25:158-61.

87. Joukhadar R, Wockel A, Herr D, Paulus V, Radosa J, Hamza A, et al. Challenges of Longevity: Safety of Vaginal and Laparoscopic Urogynecological Procedures in Septuagenarians and Older Patients. Biomed Research International. 2016;2016:9.

88. Inoue Y, Saiura A, Sato T, Ishizawa T, Arita J, Takahashi Y, et al. Laparoscopic pancreatoduodenectomy combined with a novel self-assessment system and feedback discussion: a phase 1 surgical trial following the IDEAL guidelines. Langenbecks Archives of Surgery. 2016;401(8):1123-30.

89. Greco F, Pini G, Alba S, Altieri VM, Verze P, Mirone V. Minilaparoendoscopic Single-site Pyeloplasty: The Best Compromise Between Surgeon's Ergonomy and Patient's Cosmesis (IDEAL Phase 2a). European Urology Focus. 2016;2(3):319-26.

90. Bus MTJ, de Bruin DM, Faber DJ, Kamphuis GM, Zondervan PJ, Laguna-Pes MP, et al. Optical Coherence Tomography as a Tool for In Vivo Staging and Grading of Upper Urinary Tract Urothelial Carcinoma: A Study of Diagnostic Accuracy. Journal of Urology. 2016;196(6):1749-55.

91. Kroeze SGC, Agenant M, Jonges GN, Stein T, Bosch J. Clinical efficacy of bipolar radiofrequency ablation of small renal masses. World Journal of Urology. 2015;33(10):1535-40.

92. del Val ID, Loureiro C, McCulloch P. The IDEAL prospective development study format for reporting surgical innovations. An illustrative case study of robotic oesophagectomy. Int J Surg. 2015;19:104-11.

93. Barski D, Gerullis H, Ecke T, Varga G, Boros M, Pintelon I, et al. Repair of a vesico-vaginal fistula with amniotic membrane - Step 1 of the IDEAL recommendations of surgical innovation. Central European Journal of Urology. 2015;68(4):459-61.

94. Barentsz MW, Verkooijen HM, Pijnappel RM, Fernandez MA, van Diest PJ, van der Pol CC, et al. Sentinel lymph node localization with contrast-enhanced ultrasound and an I-125 seed: An ideal prospective development study. Int J Surg. 2015;14:1-6.

95. Valerio M, Dickinson L, Ali A, Ramachandran N, Donaldson I, Freeman A, et al. A prospective development study investigating focal irreversible electroporation in men with localised prostate cancer: Nanoknife Electroporation Ablation Trial (NEAT). Contemporary Clinical Trials. 2014;39(1):57-65.

96. Sood A, Ghani KR, Ahlawat R, Modi P, Abaza R, Jeong W, et al. Application of the Statistical Process Control Method for Prospective Patient Safety Monitoring During the Learning Phase: Robotic Kidney Transplantation with Regional Hypothermia (IDEAL Phase 2a-b). European Urology. 2014;66(2):371-8.

97. Saglam R, Muslumanoglu AY, Tokath Z, Caskurlu T, Sarica K, Tasci AI, et al. A New Robot for Flexible Ureteroscopy: Development and Early Clinical Results (IDEAL Stage 1-2b). European Urology. 2014;66(6):1092-100.

98. Kaouk JH, Haber GP, Autorino R, Crouzet S, Ouzzane A, Flamand V, et al. A Novel Robotic System for Single-port Urologic Surgery: First Clinical Investigation. European Urology. 2014;66(6):1033-43.

99. Menon M, Abaza R, Sood A, Ahlawat R, Ghani KR, Jeong W, et al. Robotic Kidney Transplantation with Regional Hypothermia: Evolution of a Novel Procedure Utilizing the IDEAL Guidelines (IDEAL Phase 0 and 1). EUROPEAN UROLOGY. 2014;65(5):1001-9.

100. Heikens JT, Gooszen HG, Rovers MM, van Laarhoven C. Stages and Evaluation of Surgical Innovation: A Clinical Example of the Ileo Neorectal Anastomosis After Ulcerative Colitis and Familial Adenomatous Polyposis. Surgical Innovation. 2013;20(5):459-65.

101. Kroeze SGC, Huisman M, Verkooijen HM, van Diest PJ, Bosch J, van den Bosch M. Real-Time 3D Fluoroscopy-Guided Large Core Needle Biopsy of Renal Masses: A Critical Early Evaluation According to the IDEAL Recommendations. Cardiovascular and Interventional Radiology. 2012;35(3):680-5.

102. Solari V, Jawaid W, Jesudason EC. Enhancing safety of laparoscopic vascular control for neonatal sacrococcygeal teratoma. J Pediatr Surg. 2011;46(5):E5-E7.

103. Diepstraten SCE, Verkooijen HM, van Diest PJ, Veldhuis WB, Fernandez-Gallardo AM, Duvivier KM, et al. Radiofrequency-assisted intact specimen biopsy of breast tumors: critical evaluation according to the IDEAL recommendations. Cancer Imaging. 2011;11(1):247-52.

104. Blazeby JM, Blencowe NS, Titcomb DR, Metcalfe C, Hollowood AD, Barham CP. Demonstration of the IDEAL recommendations for evaluating and reporting surgical innovation in minimally invasive oesophagectomy. British Journal of Surgery. 2011;98(4):544-51.
