# Supplementary material for: Identifying and reporting modifications to surgical innovation: a systematic review of IDEAL/IDEAL-D studies
Source: BMJ Open. 2025 Jun 30;15(6):e097097. doi: 10.1136/bmjopen-2024-097097 (PMC12211834; doi:10.1136/bmjopen-2024-097097)
Supplement: Supplementary file 3 [file bmjopen-2024-097097.reviewer_comments.pdf]

PEER REVIEW HISTORY

BMJ Open publishes all reviews undertaken for accepted manuscripts. Reviewers are asked to complete a checklist review form and are provided with free text boxes to elaborate on their assessment. These free text comments are reproduced below.

ARTICLE DETAILS

Title (Provisional)

Identifying and reporting modifications to surgical innovation: a systematic review of IDEAL/IDEAL-D studies

Authors

Olivier, James; Elliott, Daisy; Avery, Kerry; Blencowe, Natalie S; Macefield, Rhiannon

---

VERSION 1 - REVIEW

Reviewer 1

Name Zhang, Long

Affiliation Peking University Third Hospital, Department of Interventional Radiology and Vascular Surgery, Wound Healing Center

Date 18-Dec-2024

COI None

To my knowledge, this is indeed the first systematic review around surgical innovation I have seen. The surgical innovation need to be pay attention. The authors have done a lot of review works. Unlike meta-analyze, there was a lack of evaluation indicators in the methodology, especially innovation, which leads to a pitty in the article.

Reviewer 2

Name Blakely, Brette

Affiliation Macquarie University Faculty of Arts, Philosophy Department

Date 20-Jan-2025

COI I also work on improving application of IDEAL framework and work closely with others who have worked on conceptual definitions on innovation and checklists to improve use of IDEAL.

Thank you for the opportunity to review this manuscript which furthers the cause of improving application and use of the IDEAL framework and therefore improving patient safety.

Overall the paper is very well conceived, executed and written. Therefore my feedback will be brief and fairly focused.

1) To give full transparency and credit where it is due, I suggest in your methods where you reference a single primary reviewer for the literature review, insert their initials as you did with the additional reviewers.

2) You might like to highlight your use of the term modifications more in the introduction as a more clear means of defining it for those less familiar with surgical innovation. For instance highlighting your use in these lines of your introduction would suffice: "Surgical innovations, for example, may often undergo incremental and iterative *modification* by surgeons as they perform more cases. Many of these *modifications* are not documented, reported or shared, as current evaluation systems are not designed to capture such changes." You might also add a word in the abstract similarly (eg "yet challenges remain concerning the definition and reporting of *incremental innovative* modifications, hindering evolution and evaluation of innovations and potentially risking patient safety. This systematic review examined IDEAL studies to identify *such* modifications and establish current practices around modification reporting to"

3) My final point is that I have worked with others who have examined the ambiguity of the term innovation and the difficulty in identifying what constitutes innovation. It struck me reading your paper about the more incremental modifications along the way that there are important parallels and that referencing these would, I suggest, strengthen your work, especially as you appear to have similar ambitions of creating a checklist which would support better identification and reporting. I refer as a starting point to this paper: Hutchison K, Rogers W, Eyers A, Lotz M. Getting Clearer About Surgical Innovation: A New Definition and a New Tool to Support Responsible Practice. Ann Surg. 2015 Dec;262(6):949-54. doi: 10.1097/SLA.0000000000001174. PMID: 25719812.

For full transparency, I have also worked on validation of the checklist they created. I believe that this is important related work and both could benefit from cross pollination of ideas.

Overall an interesting paper contributing to the continued efforts to make the IDEAL framework integrated into surgical innovation practices.

**Reviewer**	**3**
**Name**	**Godoi, Amanda**
**Affiliation**	**Cardiff University School of Medicine**
**Date**	**26-Mar-2025**

COI	None

The authors address a crucial issue in surgical research by examining how modifications to procedures, devices, and patient selection are reported, which is instrumental for improving transparency, patient safety, and the iterative refinement of surgical innovations. The review is conceptually sound, important in the current environment, and has a clear research objective and well-defined inclusion criteria. Well done to the authors for exploring an important area within surgical innovation and research. However, some areas require clarification, additional discussion, and minor revisions to enhance the manuscript's impact and clarity.

Major Comments

- The authors mention a previous review of IDEAL/IDEAL-D studies from 2019 (reference 36), in which studies from 2009 (time of the first IDEAL paper) to 2019 (time of the cited review) were sourced for this current paper. Firstly – what is the main purpose and difference of this review to the 2019 one? Were there methodological advancements in IDEAL/IDEAL-D reporting after 2019 that justify this restriction? If not, this appears arbitrary. The reasoning for the need for a more current review should be more explicit in the introduction section to clarify aims/purpose.

- The authors mention that the search was conducted for studies after 2019 due to the previous review. By limiting the search to post-2019 studies, the authors risk omitting studies that may have been published earlier but were not included in the prior review. The previous review may have had different inclusion/exclusion criteria, which could lead to an incomplete dataset. Relying on a single review to justify exclusion criteria can introduce bias, particularly if that review had limitations, gaps, or an incomplete search strategy. If the prior review did not focus specifically on modification reporting, relevant studies from earlier years might have been missed.

- As a suggested improvement, the authors should remove the arbitrary 2019 restriction and instead conduct a comprehensive search covering all years since the establishment of the IDEAL framework (2009 onward). If they wish to incorporate findings from the previous review, they should use it as a supplementary data source rather than as a justification for excluding pre-2019 studies. If there is a compelling reason for restricting the timeframe (e.g., a methodological shift in IDEAL reporting), the authors should clearly justify this decision.

- Another critical issue is the risk of selection bias due to overly restrictive filtering criteria. The authors limit inclusion to studies that 1) cited one of the 13 IDEAL papers and 2) explicitly mention "IDEAL" or "IDEAL-D" in the title or abstract, potentially excluding relevant studies that discuss these frameworks within the full text. This approach reduces the sensitivity of the search and may result in an incomplete dataset.

- A more rigorous approach would involve 1) full-text screening of most studies citing the 13 key IDEAL/IDEAL-D papers to determine their relevance if the topic fits most of the inclusion

criteria, rather than relying solely on title and abstract mentions. Additionally, 2) an additional search should be conducted in at least three databases to identify studies mentioning IDEAL/IDEAL-D in the title and abstract rather than restricting this process to citation-identified studies.

- Furthermore, backward and forward snowballing should be employed to identify additional relevant studies.

- The methodology does not address whether non-English studies were excluded or whether efforts were made to identify unpublished or grey literature, both of which could introduce language and publication bias.

- The method relies on studies citing key IDEAL/IDEAL-D papers. This could introduce bias by excluding relevant studies that align with the framework but did not explicitly cite these references. The authors should acknowledge this limitation in their discussion and discuss how it might affect the findings.

- The findings indicate variations in modification reporting across IDEAL stages, with Stage 2a being the most common. The manuscript would benefit from a discussion on why these disparities exist and how they might be addressed in future research. Could it be due to differing study designs, researcher awareness, or inherent challenges in modification reporting at later stages?

- Although 10% of studies were independently reviewed for validation, a larger proportion of double-screened records would enhance rigor. Best practices (Cochrane and PRISMA guidelines) typically recommend at least two independent reviewers for all initial screenings to reduce potential bias.

- The study aims to inform the development of a reporting checklist, yet there is limited discussion on how this checklist might be structured. More details on its potential implementation would strengthen the manuscript's practical contributions.

- The authors state they follow PRISMA guidelines and yet do not follow many of the components of the PRISMA checklist. Can they provide a justification for why so many aspects are “non-applicable” (such as risk of bias assessment)?

Minor Comments

- “Only two databases have the functionality required for reliable citation searching” – this could have been the two that the authors have chosen, but other databases (such as PubMed/MEDLINE) also have a citation searching tool.

- A flowchart detailing the study selection process would improve methodological clarity.

- There is little exploration of the qualitative analysis, with brief description of themes. This would be better explained in the results section with the inclusion of examples from the data to illustrate the development of thematic analysis. Expanding on this aspect would improve

the depth and rigor of the qualitative component, ensuring a clearer understanding of the modifications reported in IDEAL/IDEAL-D studies

I appreciate the opportunity to review this manuscript and look forward to the authors' responses.

VERSION 1 - AUTHOR RESPONSE

Reviewer 1 comments:

Reviewer: 1
Mr. Long Zhang, Peking University Third Hospital
Comments to the Author:
To my knowledge, this is indeed the first systematic review around surgical innovation I have seen. The surgical innovation need to be pay attention. The authors have done a lot of review works. Unlike meta-analyze, there was a lack of evaluation indicators in the methodology, especially innovation, which leads to a pitty in the article.

Response: Thank you for your review and comments, and we appreciate the recognition of the volume of work undertaken.

Reviewer 2 comments:

Reviewer: 2
Dr. Brette Blakely, Macquarie University Faculty of Arts
Comments to the Author:
Thank you for the opportunity to review this manuscript which furthers the cause of improving application and use of the IDEAL framework and therefore improving patient safety.

Overall the paper is very well conceived, executed and written. Therefore my feedback will be brief and fairly focused.

Response: Thank you to this reviewer for their positive and encouraging feedback.

1) To give full transparency and credit where it is due, I suggest in your methods where you reference a single primary reviewer for the literature review, insert their initials as you did with the additional reviewers.

Response: Thank you for this suggestion. This has been implemented through the methods section where relevant.

2) You might like to highlight your use of the term modifications more in the introduction as a more clear means of defining it for those less familiar with surgical innovation. For instance highlighting your use in these lines of your introduction would suffice: "Surgical innovations, for example, may often undergo incremental and iterative *modification* by surgeons as they

perform more cases. Many of these *modifications* are not documented, reported or shared, as current evaluation systems are not designed to capture such changes." You might also add a word in the abstract similarly (eg "yet challenges remain concerning the definition and reporting of *incremental innovative* modifications, hindering evolution and evaluation of innovations and potentially risking patient safety. This systematic review examined IDEAL studies to identify *such* modifications and establish current practices around modification reporting to"

Response: We thank you for these suggested changes to improve clarity for those less familiar with surgical innovation. We have altered the wording of the abstract as suggested (p.2). An additional explanation/clarification has also been included in the introduction(p.4):

> *'Surgical innovations, for example, may often undergo incremental and iterative modification <u>(for example, refinements or changes in technique)</u> by surgeons as they perform more cases.'*

3) My final point is that I have worked with others who have examined the ambiguity of the term innovation and the difficulty in identifying what constitutes innovation. It struck me reading your paper about the more incremental modifications along the way that there are important parallels and that referencing these would, I suggest, strengthen your work, especially as you appear to have similar ambitions of creating a checklist which would support better identification and reporting. I refer as a starting point to this paper: Hutchison K, Rogers W, Eyers A, Lotz M. Getting Clearer About Surgical Innovation: A New Definition and a New Tool to Support Responsible Practice. Ann Surg. 2015 Dec;262(6):949-54. doi: 10.1097/SLA.0000000000001174. PMID: 25719812.n

Response: Thank you for this suggestion. We are very familiar with the high-quality research from your colleagues and its relevance to our own work. We are grateful for the opportunity to update our manuscript to reference this work, and apologise that this had previously been omitted. Our research group regularly utilise findings from this paper, and we have aligned recognition of the ambiguity and difficulty surrounding the term innovation. We have now cited the Hutchinson et al 2015 paper in the introduction (p.5) as well as expanded on the relevance to our work in the discussion (p. 18) as follows:

> *'It has previously been shown that surgeons often struggle to distinguish between innovation and variation which has facilitated work on defining surgical innovation25. A tool has been developed to define innovation and enable surgeons to identify innovations and distinguish them from variations (or modifications)24,56. The checklist that is being developed from this review will complement this tool by clarifying how to identify and report modifications.'*

For full transparency, I have also worked on validation of the checklist they created. I believe that this is important related work and both could benefit from cross pollination of ideas.

Overall an interesting paper contributing to the continued efforts to make the IDEAL framework integrated into surgical innovation practices.

Response: Thank you for your review and helpful comments. We appreciate the recognition of the importance of this work.

Reviewer 3 comments:

Reviewer: 3
Miss Amanda Godoi, Cardiff University School of Medicine
Comments to the Author:
The authors address a crucial issue in surgical research by examining how modifications to procedures, devices, and patient selection are reported, which is instrumental for improving transparency, patient safety, and the iterative refinement of surgical innovations. The review is conceptually sound, important in the current environment, and has a clear research objective and well-defined inclusion criteria. Well done to the authors for exploring an important area within surgical innovation and research. However, some areas require clarification, additional discussion, and minor revisions to enhance the manuscript's impact and clarity.

Response: We would like to thank this reviewer for their positive feedback. We feel that implementing their comments and suggestions has further improved the manuscript.

Major Comments

1) The authors mention a previous review of IDEAL/IDEAL-D studies from 2019 (reference 36), in which studies from 2009 (time of the first IDEAL paper) to 2019 (time of the cited review) were sourced for this current paper. Firstly – what is the main purpose and difference of this review to the 2019 one? Were there methodological advancements in IDEAL/IDEAL-D reporting after 2019 that justify this restriction? If not, this appears arbitrary. The reasoning for the need for a more current review should be more explicit in the introduction section to clarify aims/purpose.

Response: Thank you for raising this and allowing us to explain this in more detail. We apologise for any confusion caused and agree that our manuscript could be clearer in defining the difference between the previous and current review. For clarity, the main purpose of the earlier review of IDEAL/IDEAL-D studies (published in 2020) was to examine and classify what types of outcomes (for example, procedure success, unanticipated events, surgeons' and patients' experiences) were selected, measured and reported in primary literature on innovative and invasive surgical procedures and devices. Whilst one of the categories of outcomes identified in this previous review was modifications, it did not explore modifications in any further detail. The current review aimed to examine modification reporting practices in detail. For this reason, we included all the studies identified in the earlier review and undertook new searches to identify more recent studies of innovation published after the earlier review (2019 to 2023). We followed the same methodology and search strategy to identify studies of surgical innovation (i.e. IDEAL studies) as performed in the first review. This resulted in a total of 104 included studies published between 2009 and 2023 to examine modification reporting in-depth. There have been several important methodological advancements and key publications from the IDEAL collaboration since the previous review. Therefore, this current review included citation searches for 3 new key IDEAL papers and the original ten key IDEAL papers.

Unlike previous reviews that focused on outcomes or general IDEAL reporting, our review examines explicitly how modifications to surgical innovations are reported, a topic not previously addressed in the literature. We have now added a sentence to the introduction to

highlight that a review aiming to explore modification reporting has not previously been undertaken (p.5):

> *'A review of surgical innovation studies focusing on modification reporting has not yet been undertaken. '*

2) The authors mention that the search was conducted for studies after 2019 due to the previous review. By limiting the search to post-2019 studies, the authors risk omitting studies that may have been published earlier but were not included in the prior review. The previous review may have had different inclusion/exclusion criteria, which could lead to an incomplete dataset. Relying on a single review to justify exclusion criteria can introduce bias, particularly if that review had limitations, gaps, or an incomplete search strategy. If the prior review did not focus specifically on modification reporting, relevant studies from earlier years might have been missed.

Response: We hope our response to point 1 above has helped to clarify the search process and inclusion of studies from 2009-2023, covering the entire period since the initial IDEAL publication. For further explanation, a separate supplementary file discussing the search strategy in more detail has now been created in response to the editor's suggestion (point 7), which may help to clarify this for readers. The inclusion/exclusion criteria for searches in the previous review were replicated in this review. This resulted in a total of 104 studies analysed up to July 2023. Studies predating 2019 were sourced from the prior systematic review (discussed in response to the comment above) that did not place any time limits on their searches. For studies from 2019 to 2023, the same search strategy and inclusion/exclusion criteria were applied to identify more recent publications. The only difference is the addition of the three more recent key IDEAL papers published after the previous review was conducted. This is outlined in supplementary file 1 alongside the details of the search strategy employed. We hope that this supplementary file, in addition to the PRISMA-S checklist, will allow readers to understand that the same search criteria were used for both reviews.

3) As a suggested improvement, the authors should remove the arbitrary 2019 restriction and instead conduct a comprehensive search covering all years since the establishment of the IDEAL framework (2009 onward). If they wish to incorporate findings from the previous review, they should use it as a supplementary data source rather than as a justification for excluding pre-2019 studies. If there is a compelling reason for restricting the timeframe (e.g., a methodological shift in IDEAL reporting), the authors should clearly justify this decision.

Response: Please see our response to point 2 above. We hope the supplementary file containing the detailed search strategy and our responses to comments 1) and 2) will help clarify the rationale behind the search strategy adopted for this review. We feel that this has resulted in a comprehensive search for relevant studies from 2009 onwards.

4) Another critical issue is the risk of selection bias due to overly restrictive filtering criteria. The authors limit inclusion to studies that 1) cited one of the 13 IDEAL papers and 2) explicitly mention "IDEAL" or "IDEAL-D" in the title or abstract, potentially excluding relevant studies that discuss these frameworks within the full text. This approach reduces the sensitivity of the search and may result in an incomplete dataset.

Response: Thank you for raising this. We have chosen a pragmatic approach that allows us to identify relevant primary studies reporting innovative procedures and devices. By selecting those which cite IDEAL/IDEAL-D, we have chosen those that may be considered to be following current best practices in this space. Whilst pragmatic, we agree our approach to only include studies that have cited IDEAL or IDEAL-D papers and self-identified as IDEAL/IDEAL-D studies in the title or abstract is a potential limitation. We have updated the Discussion section to acknowledge this (pp. 17- 18):

> *'Only studies that cited IDEAL/IDEAL-D and included these words within the title and/or abstract were included. This strategy was employed as it was felt that those authors actively engaging with and utilising current best practices were most likely to include thorough modification reporting. It is acknowledged that studies following the IDEAL framework but not specifying this in the abstract/title or those not citing IDEAL at all would not have been identified by the current search strategy, which might introduce an element of bias.'*

5) A more rigorous approach would involve 1) full-text screening of most studies citing the 13 key IDEAL/IDEAL-D papers to determine their relevance if the topic fits most of the inclusion criteria, rather than relying solely on title and abstract mentions. Additionally, 2) an additional search should be conducted in at least three databases to identify studies mentioning IDEAL/IDEAL-D in the title and abstract rather than restricting this process to citation-identified studies.

Response: Thank you for your comments. We recognise the merit in these suggestions. However, full-text screening of all studies citing the 13 IDEAL/IDEAL-D papers would not have been pragmatic (due to the sheer volume of records before this filtering step). Similarly, a search for studies mentioning IDEAL/IDEAL-D in the title and abstract (without restricting to those identified through citation searches) would also yield an unmanageable number of records for screening. Our study team is very experienced in developing search strategies and undertaking systematic reviews, and we chose the current approach with careful consideration and consultation. We hope we have sufficiently explained the rationale for the search strategy and the comprehensive nature of the analysis/results within the manuscript and supplementary files.

6) Furthermore, backward and forward snowballing should be employed to identify additional relevant studies.

Response: Thank you for this suggestion. In designing our systematic review, our primary objective was to capture studies where authors explicitly cited the IDEAL framework and were likely to report on surgical innovation modifications. For this reason, we focused our search strategy on citation tracking of key IDEAL publications and included studies that mentioned "IDEAL" in the title or abstract. We considered snowballing methods, but ultimately decided not to employ them for two main reasons. Firstly, given our specific inclusion criteria (studies that directly cited the IDEAL framework), we anticipated that snowballing would unlikely yield additional unique, relevant studies not already captured by our citation search. While snowballing is a valuable technique in broader literature reviews, we considered it unlikely to identify additional studies that met our specific inclusion criteria and would have introduced significant resource implications.

7) The methodology does not address whether non-English studies were excluded or whether efforts were made to identify unpublished or grey literature, both of which could introduce language and publication bias.

Response: We thank the reviewer for highlighting this and apologise for not addressing this in the manuscript. We excluded non-English studies due to a need to be pragmatic and our limited resources for accurate manuscript translation. We acknowledge that this could have implications for bias. In our search results, however, only three non-English records citing IDEAL were identified and excluded, a small proportion of the identified studies. We chose not to identify unpublished or grey literature because the review aimed to explore modification reporting in published studies of innovative and invasive surgical procedures and devices, to examine current practices surrounding publication. Findings have informed the development of a reporting checklist that applies to primary published literature, which will further be developed/tested through qualitative interviews with key stakeholders (e.g. surgeons, methodologists, trialists, industry professionals and governance experts) to ensure comprehensibility of the checklist and that it is relevant to current practices. We were reassured that we could include a total of 104 studies that covered a broad range of innovations across multiple specialities.

We have now highlighted the absence of non-English publications and grey literature as a limitation in the discussion section (p.17):

> *'Non-English language publications and grey literature were not included for pragmatic reasons due to resource limitations. Therefore some potentially relevant studies could have been missed.'*

8) The method relies on studies citing key IDEAL/IDEAL-D papers. This could introduce bias by excluding relevant studies that align with the framework but did not explicitly cite these references. The authors should acknowledge this limitation in their discussion and discuss how it might affect the findings.

Response: Thank you for this suggestion. We have further clarified this within the discussion section by adding that this may introduce an element of bias (p.17):

> *'It is acknowledged that studies following the IDEAL framework but not specifying this in the abstract/title or those not citing IDEAL at all would not have been identified by the current search strategy, which might introduce an element of bias.'*

9) The findings indicate variations in modification reporting across IDEAL stages, with Stage 2a being the most common. The manuscript would benefit from a discussion on why these disparities exist and how they might be addressed in future research. Could it be due to differing study designs, researcher awareness, or inherent challenges in modification reporting at later stages?

Response: We agree that this is an important finding and discussion point. We have clarified this and expanded upon it throughout the discussion section. Additionally, several sentences have been added to discuss this further (p.16):

‘The IDEAL framework recognises the importance of reporting modifications, particularly in stage 2a, but lacks clarity over how this should be done in practice7,17. This emphasis on modification reporting in early innovation may be reflected in the frequency of modification reporting in Stage 2a studies within this review. The observed variation in modification reporting in other IDEAL stages may be partly due to a lack of guidance on identifying and reporting modifications in surgical innovation, particularly in non-stage 2a studies19. The studies included in this review demonstrated inconsistent approaches to implementing and using IDEAL guidance across all stages, which aligns with findings from previous reviews on the uptake of the IDEAL framework12,51.’

10) Although 10% of studies were independently reviewed for validation, a larger proportion of double-screened records would enhance rigor. Best practices (Cochrane and PRISMA guidelines) typically recommend at least two independent reviewers for all initial screenings to reduce potential bias.

Response: Thank you for your comment. To clarify, we undertook independent double screening for 100% of the records to reduce potential bias for the full text stage of screening following best practices recommended by Cochrane and PRISMA. Independent double screening of 10% of records was undertaken in the title/abstract screening stage, carried out for a random sample of abstracts by alphabetising the records by title and selecting every tenth study from the 1071 records. Full agreement/concordance between reviewers for this sample was observed, so further double-screening of the remainder of the 1071 was not warranted. Cochrane recommends double screening for the full-text, but states that single reviewer screens at the abstract stage can be undertaken (‘it is acceptable that this initial screening of titles and abstracts is undertaken by only one person’ – Cochrane handbook, Version 6.5, 2024, section 4.6.4). We took an inclusive approach at the title/abstract stage, whereby any studies that were ‘maybe’ went through to the full text screening stage for double review (to avoid potential incorrect exclusion). These strategies were employed following best practice guidelines to minimise potential bias.

11) The study aims to inform the development of a reporting checklist, yet there is limited discussion on how this checklist might be structured. More details on its potential implementation would strengthen the manuscript's practical contributions.

Response: Thank you for this comment. This systematic review was the first component in a broader package of work looking to develop and refine a reporting checklist. It was critical to understand how modifications are reported and assess the variation in practice. Whilst we look forward to being able to describe and disseminate this work in future publications, the current manuscript aims to focus on the review and its findings. The development of the checklist and details on how it is structured are subsequent steps and will be reported in due course. The discussion section highlights how this checklist will fit with current literature and guidance P.18).

12) The authors state they follow PRISMA guidelines and yet do not follow many of the components of the PRISMA checklist. Can they provide a justification for why so many aspects are “non-applicable” (such as risk of bias assessment)?

Response: Thank you for reviewing our PRISMA checklist and your comments. We have commented on the absence of a risk of bias assessment of the included studies within the manuscript: *'Given the exploratory nature of this review, a risk of bias or quality assessment for the included studies was not undertaken.'* Other papers looking at surgical innovation studies were also unable to undertake such assessments due to the heterogeneity of the data and the exploratory nature of their reviews. We have now referenced some of these studies in the manuscript with the addition of the following text (p.10):

> *'Given the exploratory nature of this review, a risk of bias or quality assessment for the included studies was not undertaken, similar to that of other exploratory reviews of surgical innovation studies40,50'*

We have also now included a PRISMA-S checklist with the updated manuscript, which highlights further PRISMA components that have been followed for the reporting of this study.

Minor Comments

13) "Only two databases have the functionality required for reliable citation searching" – this could have been the two that the authors have chosen, but other databases (such as PubMed/MEDLINE) also have a citation searching tool.

Response: Thank you for your comment. Whilst other databases have some citation functionality, these are limited compared to Scopus and WoS. Several papers have highlighted the benefits of these databases for citation searches. We have clarified this within the methods and discussion sections in the revised manuscript (p.6 + p.17), with additional references/citations.

14) A flowchart detailing the study selection process would improve methodological clarity.

Response: A PRISMA flowchart has been included as a separate file (as per the journal requirements). This outlines the study selection process, including the identification, screening and results of the searches. Please see Figure 1 for details.

15) There is little exploration of the qualitative analysis, with brief description of themes. This would be better explained in the results section with the inclusion of examples from the data to illustrate the development of thematic analysis. Expanding on this aspect would improve the depth and rigor of the qualitative component, ensuring a clearer understanding of the modifications reported in IDEAL/IDEAL-D studies

Response: Thank you for this suggestion. The manuscript did not intend to report a detailed qualitative analysis, instead we focus on a descriptive summary of the findings to present a succinct overview of current practices in reporting modifications. The qualitative findings do not differ from those reported in this manuscript and were not included to keep the manuscript succinct. We provide a brief description of the qualitative component and the identified themes for transparency of our wider methodology. This review is the first step in a broader package of work aiming to produce standardised guidance on how to report modifications in studies reporting innovative devices and procedures. The detailed qualitative findings from this review will be combined with findings from subsequent in-depth stakeholder interviews to optimise the reporting checklist. We intend to report the thematic synthesis findings from this review in a

doctoral thesis (which will be freely available online) and subsequent publications. We have made this clearer in the manuscript with additional text in the discussion section (p.18):

> *'The detailed qualitative findings from this review will be presented in future publications when synthesised and combined with findings from subsequent in-depth stakeholder interviews.'*

I appreciate the opportunity to review this manuscript and look forward to the authors' responses.

Response: Thank you for your detailed review, and we hope that our responses have facilitated further enhancement of the manuscript.

VERSION 2 - REVIEW

**Reviewer**	**3**
**Name**	**Godoi, Amanda**
**Affiliation**	**Cardiff University School of Medicine**
**Date**	**18-Jun-2025**
**COI**	

Dear authors, thank you for responding to our comments thoroughly and demonstrating a genuine effort to revise the manuscript in light of our critiques.

My main concerns were regarding the search strategy and approaches used for study identification. The authors have now more clearly specified their screening methodology in the supplementary material, which is appreciated. Thank you for also explicitly stating your limitations in the discussion section in light of the comments.

While I appreciate the rationale provided, I remain concerned that the absence of a search update between July 2023 and the present may limit the currency and "state of the art" aspect of this review, particularly in a rapidly evolving field such as surgical innovation.

Given that the review includes 13 IDEAL/IDEAL-D studies, a targeted update (e.g., solely identifying IDEAL/IDEAL-D-related publications in PubMed from mid-2023 to 2025) would not pose a substantial burden and would likely involve screening fewer than 500 studies (considerably lower than traditional screening processes). Such a step could feasibly strengthen the timeliness and comprehensiveness of the conclusions.

That said, I acknowledge you have now appropriately acknowledged this limitation within the manuscript and revised the language accordingly. Provided the Editor is satisfied with the current framing and transparency regarding the search window, I am content to proceed with acceptance, recognising the value of the review as a foundational and exploratory contribution.
